# The Physical Basis of Coordinated Tissue Spreading in Zebrafish Gastrulation

**DOI:** 10.1016/j.devcel.2017.01.010

**Published:** 2017-02-27

**Authors:** Hitoshi Morita, Silvia Grigolon, Martin Bock, S.F. Gabriel Krens, Guillaume Salbreux, Carl-Philipp Heisenberg

**Affiliations:** 1 Institute of Science and Technology Austria, Am Campus 1, 3400 Klosterneuburg, Austria; 2 The Francis Crick Institute, 1 Midland Road, London NW1 1AT, UK; 3 Max-Planck-Institute for the Physics of Complex Systems, Nöthnitzer Straße 38, 01187 Dresden, Germany

**Keywords:** active fluid description of tissue spreading, radial cell intercalation, surface cell expansion, interfacial tension, gastrulation, zebrafish

## Abstract

Embryo morphogenesis relies on highly coordinated movements of different tissues. However, remarkably little is known about how tissues coordinate their movements to shape the embryo. In zebrafish embryogenesis, coordinated tissue movements first become apparent during “doming,” when the blastoderm begins to spread over the yolk sac, a process involving coordinated epithelial surface cell layer expansion and mesenchymal deep cell intercalations. Here, we find that active surface cell expansion represents the key process coordinating tissue movements during doming. By using a combination of theory and experiments, we show that epithelial surface cells not only trigger blastoderm expansion by reducing tissue surface tension, but also drive blastoderm thinning by inducing tissue contraction through radial deep cell intercalations. Thus, coordinated tissue expansion and thinning during doming relies on surface cells simultaneously controlling tissue surface tension and radial tissue contraction.

## Highlights

• The blastoderm actively spreads over the yolk cell during zebrafish doming
• Epithelial surface cell expansion triggers doming by reducing tissue surface tension
• Radial deep cell intercalations facilitate doming by radially contracting the tissue
• Epithelial surface cell expansion triggers intercalation of underlying deep cells

## Graphical Abstract

**Figure:**
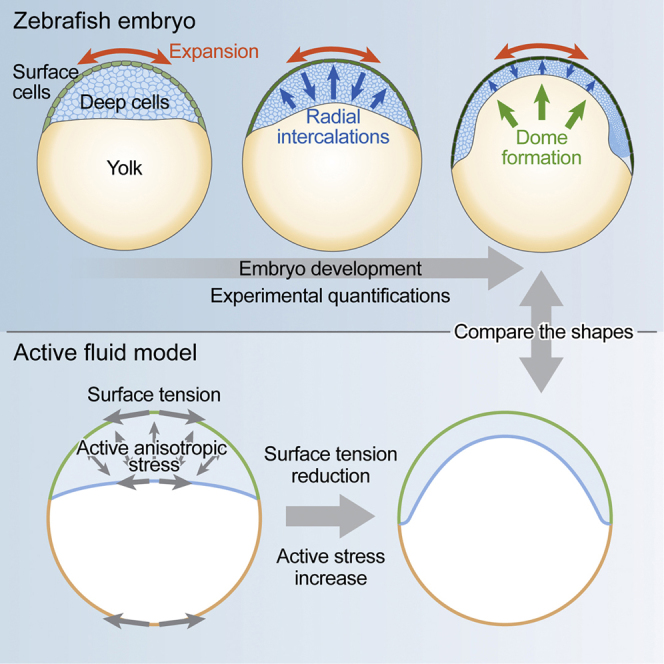

## Teaser

Morita et al. combine computational modeling and experiments to show that at zebrafish gastrulation onset, epithelial surface cells coordinate blastoderm thinning and expansion for its spread over the yolk cell. Active surface cell expansion simultaneously triggers tissue expansion by reducing tissue surface tension and tissue thinning by inducing deep cell radial intercalations.

## Introduction

Coordinated motion of cells and tissues is a common feature of tissue and embryo morphogenesis in development (Solnica-Krezel, 2005). Although the fundamental processes driving cellular rearrangements within a tissue are beginning to be unraveled (Guillot and Lecuit, 2013, Heisenberg and Bellaïche, 2013, Walck-Shannon and Hardin, 2014, Wallingford, 2012), very little is yet known about how different tissues interact to coordinate their movements at the embryo scale.

Tissue spreading, the simultaneous expansion and thinning of a tissue, represents a key morphogenetic event in various developmental and disease-related processes, such as gastrulation and wound healing. While spreading of simple epithelial cell sheets is typically achieved by epithelial cell expansion and/or division, spreading of complex multilayered tissues often involves coordinated expansion of epithelial surface cells and radial intercalations of more mesenchymal deep cells. In *Xenopus* gastrulation, for instance, the blastocoel roof spreads by radial intercalation of deep cells on the basal side of the overlying superficial epithelial cells, which in turn undergo pronounced expansion (Keller, 1978). Likewise in mouse embryogenesis, epidermal spreading has recently been associated with expansion of superficial cells and radial intercalation of deep cells (Panousopoulou et al., 2016). Yet, how surface cell expansion and radial deep cell intercalation function together to trigger tissue spreading remains unclear.

**Figure 1:**
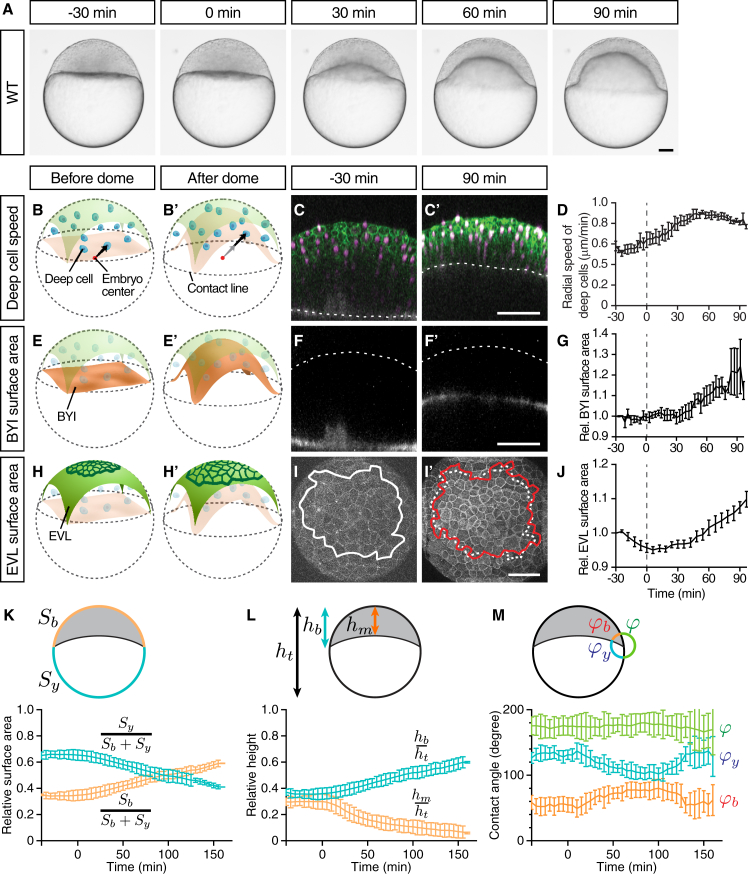
Doming Is Associated with EVL Cell Expansion and Radial Deep Cell Intercalations (A) Bright-field images of a zebrafish WT embryo at sequential stages from the pre-doming stage (−30 min) to the end of doming (+90 min). (B, B′, E, E′, H, and H′) Schematic representation of a zebrafish embryo before and after doming illustrating deep cell radial movement (B) and (B′), BYI upward bulging (E) and (E′), and EVL expansion (H) and (H′). BYI, blastoderm-to-yolk cell interface. Arrows, radial movement of deep cells. (C, C′, F, F′, I, and I′) Confocal images of the blastoderm before the onset (−30 min) and after completion of doming (+90 min) where membrane, green in (C) and (C′) and white in (I) and (I′); nuclei, magenta in (C) and (C′); and BYI, white in (F) and (F′) were labeled by membrane-targeted GFP (mem-GFP), H2A-mCherry, and fluorescent dextran, respectively. Dashed lines mark the BYI in (C) and (C′) or outer surface of the blastoderm in (F) and (F′). Solid lines in (I) and (I′) outline measured surface area, and dashed line in (I′) marks the measured surface area at −30 min (I). (D) Average deep cell speed along the radial direction of the embryo plotted as a function of time during doming. (G) Relative BYI surface area measured within the observed region of the embryo and plotted as a function of time during doming. (J) Relative EVL surface area measured for a continuous patch of cells within the observed region of the embryo and plotted as a function of time during doming. (K–M) Geometrical parameters of WT embryos during doming with relative surface area (K) (*S*_*b*_, entire blastoderm surface area; *S*_*y*_, entire yolk surface area), relative height (L) (*h*_*b*_, height of the blastoderm between animal pole and contact line; *h*_*m*_, height of the blastoderm at the center of the embryo; *h*_*t*_, total height of the embryo) and contact angles (M) (*φ*_*b*_, angle between EVL and BYI; *φ*_*y*_, angle between BYI and yolk membrane; *φ*, angle between yolk membrane and EVL) quantified from bright-field embryo images. n = 6 embryos. Error bars, ±SEM (D), (G), and (J) and ±SD (K–M). Scale bars, 100 μm. Time point 0 min always indicates the beginning of doming recognizable by an upward bulging of the BYI. Embryo images are lateral views with the animal pole up unless otherwise stated. (I) and (I′) are animal pole views. See also Figure S1 and Movie S1.

At the onset of zebrafish gastrulation, the blastoderm starts spreading over the spherical yolk cell in a movement called doming (Figure 1A and Movie S1; Lepage and Bruce, 2010). The blastoderm is composed of a simple squamous epithelial surface cell layer, named the enveloping layer (EVL), and mesenchymal cells positioned below this layer, which form the pool of germ layer progenitor cells and are named deep cells. Doming has predominantly been associated with deep cells undergoing radial intercalations (Lepage and Bruce, 2010, Warga and Kimmel, 1990). In addition, upward pushing by the yolk cell (Wilson et al., 1995) and epithelial integrity of surface cells (Lepage et al., 2014) have been involved. Still, how these different processes are spatiotemporally coordinated during doming, and how they contribute to the force-generating processes underlying tissue shape changes during doming is only poorly understood.

Here, we have used a combination of theory and experiments to unravel the fundamental force-generating processes driving doming. We show that tissue spreading during doming is driven by two distinct yet interdependent force-generating processes: epithelial surface cells actively expanding by reducing their surface tension, and deep cells undergoing radial cell intercalations and thus generating anisotropic active stress within the bulk of the tissue. We further show that active surface cell expansion not only triggers tissue expansion but also induces active radial deep cell intercalations required for homogeneous tissue thinning during spreading.

## Results

### Doming Is Associated with Radial Deep Cell Intercalations and EVL Cell Expansion

To identify the fundamental force-generating processes driving doming, we first analyzed cellular rearrangements and shape changes during doming. Consistent with previous observations (Bensch et al., 2013), we found that deep cells underwent extensive intercalations along the radial axis of the blastoderm without invading the overlying EVL (Figures 1B–1D). Deep cell intercalations were accompanied by the blastoderm starting to spread over the yolk cell, recognizable by an upward bulging of the blastoderm-to-yolk cell interface (BYI; Figures 1E–1G), commonly termed dome formation (Kimmel et al., 1995). Notably, we also observed that EVL cells began to spread along the blastoderm surface by expanding their surface area at the same time as doming was initiated (Figures 1H–1J). The onset of all of these different morphogenetic processes roughly coincided with the onset of doming as defined by the upward bulging of the BYI, pointing to the possibility that they are functionally linked. Interestingly, EVL spreading became apparent slightly later than the onset of radial deep cell motility, suggesting that these processes might not be directly coupled. Together, these observations indicate that doming involves radial intercalations of deep cells, upward bulging of the BYI, and expansion of EVL cells.

To determine how those cellular processes relate to overall changes in embryo geometry, we segmented images of embryo cross-sections over the course of doming. Assuming rotational symmetry of the embryo around the animal-vegetal (AV) axis, we then quantified several geometrical measurements characterizing the embryo shape (Figures 1K–1M). Specifically, we quantified the extent of EVL surface area expansion by recording changes in the ratio of blastoderm and yolk surface area to the total embryo surface area (Figure 1K). Furthermore, we quantified the extent of blastoderm thinning by measuring the ratio of blastoderm height along the AV axis to the total embryo height (Figure 1L). These measurements showed that during the course of doming the EVL surface area expands and the blastoderm height shrinks (Figures 1K and 1L). Finally, we also quantified the contact angles between the blastoderm surface, yolk cell surface, and BYI at the contact line (De Gennes et al., 2004) where these three interfaces meet. Interestingly, we found that the angle between BYI and blastoderm surface was not decreasing, as expected if the BYI would homogeneously bulge upward, but in fact was increasing by about 30° within the first 20 min of doming (Figure 1M). This suggests that the BYI undergoes distinct shape changes during doming with its center bulging upward and its edge moving in the opposite direction bulging downward.

### Doming Movements Can Be Simulated in Physical Models Based on Changes in Blastoderm Surface Tension and Anisotropic Radial Stress

**Figure 2:**
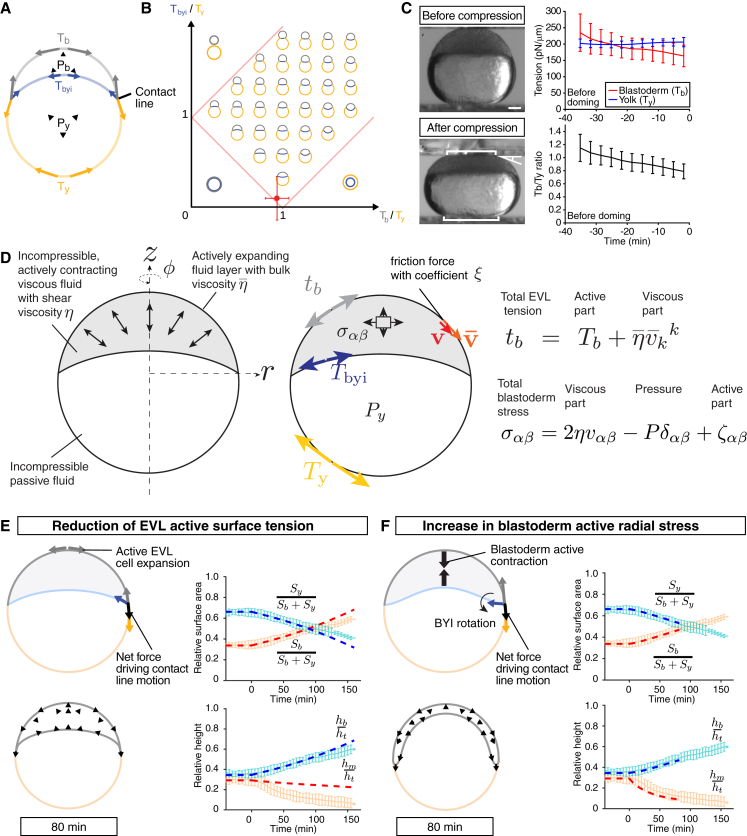
Physical Description of Doming Based on Changes in Blastoderm Surface Tension and Radial Stress (A) Schematic of the surface tension model for describing embryo shapes at the onset of doming. The ratio of volumes of blastoderm and yolk is fixed, and the blastoderm, yolk, and BYI interfaces are subjected to surface tensions. (B) Phase diagram of equilibrium shapes predicted by the surface tension model of the embryo as a function of the surface tension ratios $T_{b}/T_{y}$ and $T_{\mathrm{byi}}/T_{y}$. Red dot, predicted ratio of surface tensions before doming from analysis of embryo shape. (C) Measurement of blastoderm (*T*_*b*_) and yolk cell surface tension (*T*_*y*_) in WT embryos at the onset of doming using tissue tensiometry. Embryos were compressed by 20% of their initial uncompressed height. Panels on the left are bright-field images of a WT embryo before (top) and after compression (bottom). Brackets, contact areas. Kinked line, contact angle. Panels on the right show *T*_*b*_ and *T*_*y*_ (top) and $T_{b}/T_{y}$ (bottom) as a function of time after compression. n = 4 embryos. Error bars, ±SD. Scale bar, 100 μm. (D) Schematic of the dynamic model of doming. The blastoderm is represented by an incompressible viscous fluid with shear viscosity η, subjected to an anisotropic, radially oriented active internal stress with magnitude ζ. The EVL is represented by a compressible fluid with bulk surface viscosity $\bar{\eta }$, subjected to an active surface tension $T_{b}$. The yolk is represented by an incompressible fluid with negligible viscosity and a surface tension $T_{y}$. (E and F) Two hypothetical scenarios for doming where either EVL surface tension reduction results in an imbalance of forces at the contact line, driving EVL surface expansion (E) or active radial contraction of the deep cell layer drives blastoderm thinning (F). In (F) blastoderm deformation leads to a rotation of the BYI tangent vector at the contact line, resulting in a net force at the contact line driving EVL surface expansion. Left panels in (E) and (F) are schematic representations of the different mechanisms on which the simulations are based on and the simulated embryos shapes at 80 min of doming. Black arrows outline blastoderm velocity field. Right panels in (E) and (F) are plots of embryo surface area and height as a function of time during doming with pale blue and red curves showing the experimental measurements, and dashed red and blue thick lines showing simulation results. Simulations parameters are specified in Table S1C. Simulations in (F) were stopped when the angle between the EVL and the YSL approached a zero value. Error bars, ±SD. See also Figure S2 and Movie S2.

To unravel the force-generating processes underlying tissue shape changes during doming, we first developed a physical model of doming based on the balance of forces exerted in the blastoderm, yolk, and EVL. To start with, we sought to explain the shape of the embryo at the onset of doming. We assumed that the surfaces of the yolk cell, blastoderm, and the BYI are under tension, and that deep cells can be treated as a fluid material, such that the pressure in the tissue is uniform in the absence of deep cell flows. These hypotheses are consistent with the circular shape of the blastoderm and yolk cell surfaces, and the BYI prior to doming (Figure 2A), and our observation that blastoderm explants round up in vitro (Schötz et al., 2008). We also assumed, based on our experimental measurements (Figure S1), that the ratio of yolk to blastoderm volume was constant. The balance of forces at the contact line and of pressures at different interfaces then sets the shape of the embryo as a function of the ratios of the interfacial tensions of the yolk (*T*_y_), blastoderm (*T*_b_), and BYI (*T*_byi_) (Figure 2A). We found that a ratio of *T*_b_*/T*_y_ = 0.94 and *T*_byi_*/T*_y_ = 0.11 gave rise to predicted embryo shapes closely matching the experimental observations (Figure 2B). Interestingly, the ratio of *T*_b_*/T*_y_ is close to the critical boundary defining whether the BYI bulges upward or downward (Figure 2B), likely reflecting the fact that the average surface tension of the embryo is quite large (thus its overall spherical shape), and that, consequently, even small changes in the EVL and yolk surface tensions can drive either EVL contraction or expansion.

To verify this prediction about interfacial tension ratios and thus validate our general modeling approach, we simultaneously measured the surface tensions of blastoderm and yolk cell during a period of 30 min prior to doming by compressing embryos using a tissue tensiometer (Foty et al., 1994). We applied a 20% deformation of the embryo along its AV axis and measured the force exerted by the embryo on the plate and the deformed shape of the embryo. To obtain the surface tension of the yolk and blastoderm from these measurements, we assumed as before that the surfaces of the yolk cell and blastoderm and the BYI are under tension, and that deep cells and the yolk material can be treated as fluids. We also took into account gravity forces exerted on the embryo due to its mass. The mass of the embryo was measured by performing sinking experiments and recording the steady-state sinking velocity of the embryo (Methods S1). With this we could then estimate the yolk surface tension *T*_y_ = 206 ± 13 pN/μm (n = 4) and the blastoderm surface tension *T*_b_ = 167 ± 33 pN/μm (n = 4) (Figures 2C and S2A), closely matching the values for the ratio of *T*_b_*/T*_y_ predicted by our model (Figure 2B).

Having verified our model prediction about interfacial tension ratios and thus validated our general modeling approach of representing the embryo as a fluid material with surface tension, we then developed a dynamic model of the mechanics of the embryo during doming in order to identify the underlying force-generating processes (Figures 2D and S2B–S2E). In this model, we assumed that tensions at the yolk cell surface and BYI are constant, consistent with our observations that neither yolk cell surface tension (Figure S3B) nor actomyosin distribution at the BYI (Figure S3C) displayed any major alterations during the doming process. We further assumed that the total surface tension of the blastoderm $t_{b}$ has a viscous contribution that is dependent on the rate of expansion of the EVL ${\bar{v}}_{i}^{i}$ and can be written as

(Equation 1) $$t_{b}=T_{b}+\bar{\eta }{\bar{v}}_{i}^{i}\;\text{,}$$

where $T_{b}$ is a constant contribution to the surface tension and $\bar{\eta }$ is the EVL bulk surface viscosity. A reduction of the surface tension $T_{b}$ then contributes to EVL expansion, at a rate which is limited by the EVL viscosity. We also assumed that deep cells could be treated as an active fluid material. For simplicity, we also took the total volume of the embryo to be fixed in our simulations (Figure S1). The stress tensor $\sigma_{\alpha \beta }$ acting within the blastoderm can then be written as

(Equation 2) $$\sigma_{\alpha \beta }=2\eta v_{\alpha \beta }-P\delta_{\alpha \beta }+\zeta_{\alpha \beta }\;\text{,}$$

where $v_{\alpha \beta }$ is the gradient of flow in the blastoderm, η is the shear viscosity of the blastoderm, and P is the pressure ensuring volume conservation of the tissue. In the stress Equation (2), we also included anisotropic active stresses in the deep cell layer $\zeta_{\alpha \beta }$, which are oriented along the radial axis of the blastoderm. We included these anisotropic active stresses to account for the potential force generation by active radial deep cell intercalations (Bensch et al., 2013), in line with previous approaches that have captured autonomous cellular force generation within a tissue by the contribution of an active stress to the total stress of the tissue (Prost et al., 2015, Ranft et al., 2010, Etournay et al., 2015, Behrndt et al., 2012). We finally assumed, based on our measurements of blastoderm and yolk viscosities (Figures S3I and S3K; Methods S1), that the yolk viscosity ($\eta_{y}∼40$ Pa s) is much smaller than the blastoderm viscosity ($\eta ∼10^{3}$ Pa s, Table S1C) and thus can be neglected. For simplicity, we also supposed that the simulated embryo has rotational symmetry around the AV axis. To solve for the blastoderm and EVL flow, we employed a finite-element method (Methods S1), where we took the size of the elements to be of the order of the cell size, such that in the region near the contact line where the blastoderm size becomes comparable with the cell size, the gradient of flow and pressure are homogeneous (Methods S1).

For blastoderm expansion, the contact line has to move vegetally. We considered two different hypothetical scenarios for the forces driving this motion. In the first scenario, there is no active radial stress generation within the deep cell layer, but EVL cells actively expand by reducing their surface tension. This results in an imbalance of forces acting on the contact line, driving its motion toward the vegetal pole (Figure 2E). In a second scenario, the EVL is not autonomously reducing its surface tension, but deep cells generate active radial stresses within the blastoderm. As a result, deep cells will flow to the margin of the blastoderm and, consequently, the BYI will deform at its margin. This deformation will cause a rotation of the BYI tangent vector at the contact line, shifting the balance of forces at the contact line and, consequently, leading to passive EVL expansion (Figure 2F).

To test which of these hypothetical scenarios describes best the experimentally observed doming movements, we performed simulations of embryo shape changes using a finite-element method, including either reduced blastoderm surface tension or active radial stress within the blastoderm (Methods S1). We found that simulations of both scenarios gave rise to blastoderm expansion reminiscent of the actual situation in vivo (Figures 2E and 2F; Movie S2). However, only simulations based on active radial stress, but not reduced surface tension, also produced thinning of the blastoderm along its AV extent comparable with the situation in vivo (Figure 2F). This initial analysis suggests that contraction of the deep cell layer constitutes a more plausible force-generating process driving embryo shape changes during doming than a reduction in blastoderm surface tension. However, contact angles were only poorly predicted in either of our simulations (Figures S2F and S2G), prompting us to closer investigate the role of radial active stress generation for doming.

**Figure 3:**
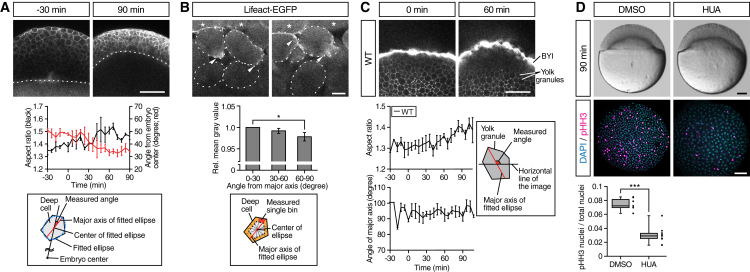
Deep Cells and Yolk Granules Change Their Shape during Doming (A) Deep cell shape changes in WT embryos. Upper panels are single plane confocal images of deep cells close to the EVL before (−30 min) and after completion of doming (+90 min). Deep cell plasma membrane was labeled by mem-RFP. Dotted lines, BYI position. Middle panel shows quantification of deep cell aspect ratio (black line, left vertical axis) and angle from embryo center (red line, right vertical axis) of deep cells in embryos from −30 to +90 min of doming. Bottom panel shows schematic of deep cell angle measurements. n = 3 embryos. Error bars, ±SEM. Scale bar, 100 μm. (B) Subcellular localization of actin in deep cells during doming. Upper panels are single plane confocal images of deep cells close to the EVL in *Tg(actb1:lifeact-EGFP)* embryos during doming. Dotted lines, plasma membrane of individual cells. Asterisks, EVL cells. Arrowheads, polarized actin localization in deep cells. Middle panel shows quantification of actin distribution in polarized deep cells (aspect ratio ≥1.6) during 90 min of doming. Bottom panel shows schematic of the actin distribution analysis in deep cells within an area ≈3 μm away from the plasma membrane (orange) and subdivided in 30° bins relative to the major axis of the fitted ellipse (red). n = 3 embryos. Error bars, ±SEM; ^∗^p < 0.05; t test. Scale bar, 10 μm. (C) Yolk granule shape in intact WT embryos. Upper panels are single plane confocal images of yolk granules in embryos injected with fluorescent dextran into the yolk at 0 min (left) and +60 min (right) of doming. Lower left panels are quantification of yolk granule aspect ratio (top) and angle of major axis (bottom) from −30 to +108 min of doming. Lower right panel shows schematic of measured angle in yolk granules. n = 4 embryos. Error bars, ±SEM. Scale bar, 100 μm. (D) Doming in WT embryos and embryos treated with hydroxyurea and aphidicolin (HUA) to block cell divisions. Upper panels are bright-field images of control (left) and HUA-treated embryos (right) after completion of doming (+90 min). Lower panels are confocal images of control (left) and HUA-treated embryos (right) immunostained for the mitotic marker phosphorylated histone H3 (pHH3; magenta) at the onset of doming (0 min) from animal pole view. All nuclei were labeled by DAPI (cyan). Lower left plot shows ratio of pHH3-positive nuclei to total nuclei number. Boxplots represent 25%, median, 75%, and 95%. ^∗∗∗^p < 0.001; t test. n = 7 embryos (DMSO) and 10 embryos (HUA). Scale bar, 100 μm. See also Figure S2 and Movie S3.

To determine whether and how anisotropic stresses are generated within the deep cell layer during doming, we analyzed the shape and subcellular localization of actin in deep cells during doming. We found that deep cells were preferentially elongated along the radial axis of the blastoderm and showed actin accumulations at their protrusive front end pointing either toward or away from the EVL (Figures 3A and 3B; Movie S3). Given that deep cells undergo radial cell intercalations (Bensch et al., 2013, Lepage and Bruce, 2010, Warga and Kimmel, 1990) but do not contract along the AV axis (Figure 3A), and that deep cell divisions are dispensable for doming (Figure 3D), radial deep cell polarization most likely leads to the generation of anisotropic radial stress within the blastoderm by triggering radial cell intercalations. To test whether stresses generated in the blastoderm are also transmitted to the yolk cell, we analyzed deformation of yolk granules at the BYI during doming. We found that with the onset of doming, yolk granules, which occupy most of the yolk cell cytoplasm below the deep cell layer, began to elongate along the radial axis (Figure 3C), indicating that stresses generated within the deep cell layer are transmitted to the yolk cell, deforming yolk granules.

### Radial Cell Intercalations Are Dispensable for Blastoderm Spreading but Required for Blastoderm Thinning during Doming

**Figure 4:**
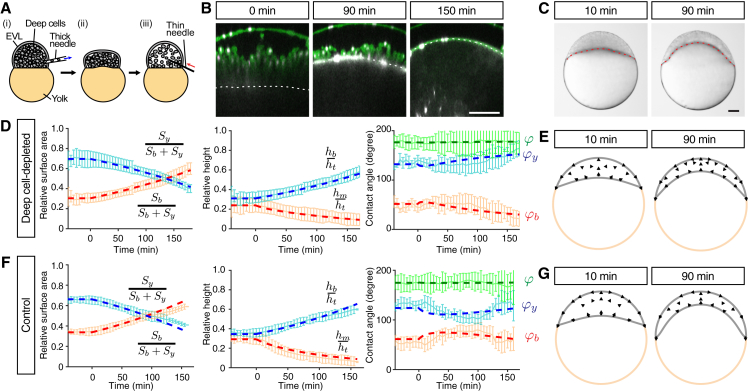
Radial Cell Intercalations Are Dispensable for Blastoderm Spreading but Required for Blastoderm Thinning during Doming (A) Schematic of the deep cell-depletion experiment. (1) Deep cells were removed with a thick needle, (2) the removal reduced blastoderm volume, and (3) the blastoderm volume was restored to its original size by injection of embryo medium. (B) Single-plane confocal images of deep cell-depleted WT embryos before (−30 min) and after completion of doming (+90 and +150 min). Plasma membrane was marked by mem-GFP in green. Nuclei were marked by H2A-mCherry in magenta. BYI was marked by dextran in white. (C) Bright-field images of deep cell-depleted embryo at the onset (+10 min) and after doming (+90 min). (D–G) Comparison of experimentally measured embryo shape changes with simulation results adjusted to reproduce experimental observations of deep cell-depleted embryos (D) and (E) and intact WT embryos (F) and (G) during doming. Simulation parameters are listed in Table S1C. Left plots are comparison of embryo surface area, height and contact angle with pale blue and red curves showing the experimental measurements, and dashed red and blue thick lines showing the simulation results. Right panels show simulated embryo shapes at 10 and 90 min of doming with black arrows marking the blastoderm velocity field. The experimental data in (F) were taken from Figures 1K–1M. n = 5 embryos (deep cell-depleted) and 6 embryos (control). Error bars, ±SD. Scale bars, 100 μm. See also Figures S3 and S4; Movie S4.

While our observations so far suggest that the deep cell layer actively contracts along the radial axis of the embryo (Figures 3A–3C), and that this contraction is in principle sufficient to trigger blastoderm expansion and thinning (Figure 2F), direct evidence for a key role of radial stress for doming is still missing. To test whether doming can occur in the absence of active radial stress within the deep cell layer, we manually removed a large portion (≈60%) of the deep cells from embryos shortly before the onset of doming (3 hr postfertilization, hpf) and replaced them with embryo medium (Danieau's solution) of approximately the same volume (Figures 4A and S3A). Unexpectedly, we found that in embryos lacking a large portion of deep cells, surface cell expansion, and upward bulging of the BYI, key features of doming, appeared relatively unaffected (Figures 4B and 4C; Movie S4). In contrast, deformation of yolk granules along the radial axis was absent in deep cell-depleted embryos (Figures S3D and S3E), suggesting that radial stress generated by deep cells within the blastoderm causes granule deformation, and that this stress is reduced upon removal of deep cells. Together, this suggests that, contrary to our initial expectations based on our simulations, active contraction of the deep cell layer along its radial axis does not constitute the main force-generating mechanism driving doming.

To determine whether active radial stress might have any function during doming, we analyzed shape changes in deep cell-depleted embryos in detail. We found that the reduction in blastoderm height and downward motion of the BYI tangent vector at the contact line was less pronounced in deep cell-depleted embryos compared with intact wild-type (WT) embryos (Figure S3F). Notably, these features of embryo shape changes were similar to results obtained by simulating doming based on reduced surface tension alone (Figure 2E). We thus speculated that doming movements might be achieved by two distinct force-generating processes: EVL cells autonomously reducing their surface tension thereby driving overall blastoderm spreading over the yolk cell, and deep cells generating radial stress within the bulk of the tissue thereby promoting homogeneous blastoderm thinning during the spreading process. To test this assumption, we performed simulations assuming a combination of reduced blastoderm surface tension and active radial stress within the deep cell layer, and no friction between the EVL and deep cells. We first aimed at independently measuring the blastoderm shear viscosity (η), using established biophysical methods (Gonzalez-Rodriguez et al., 2012). To this end, we performed compression experiments of blastoderm explants to obtain their surface tension ($T_{e}≃100\;\text{pN/μm}$, Figure S3H). We then performed fusion experiments of two explants put into contact, and monitored their shape relaxation from two spherical caps to a single sphere (Figure S3I). Finally, we performed a finite-element simulation of this fusion process (Methods S1) and found that the dynamics of shape relaxation could be explained for a blastoderm shear viscosity $\eta ≃10^{3}\;\text{Pa}\;\text{s}$. With this value in hand, we turned to WT and deep cell-depleted embryos, and adjusted the remaining parameters of our physical description to match embryo shape changes during doming. Specifically, we adjusted the viscosity of the EVL ($\bar{\eta }$), the decrease in EVL surface tension $\Delta T_{b}/T_{b}$ at the onset of doming, and the magnitude of the anisotropic active stress within the blastoderm. Importantly, our parameter search was considerably constrained by the observations that the EVL area expansion rate and timescale of blastoderm height are strongly dependent on the timescale $\bar{\eta }/\Delta T_{b}$ and η/ζ, respectively, and that the ratio of viscosities $\bar{\eta }/\eta$ influences the contact angle dynamics (Figure S4). We found that shape changes obtained in these simulations could be closely matched to experimental observations of both WT and deep cell-depleted embryos (Figures 4D–4G and Movie S4) for a reduction of EVL surface tension, $\Delta T_{b}/T_{b}$ = 25%, an EVL bulk surface viscosity of $\bar{\eta }$ ∼8 × 10^5^ pN/μm s, and a magnitude of the blastoderm anisotropic active stress of *ζ* ∼0.5 Pa (Table S1C). We also found that for accurately simulating shape changes in deep cell-depleted embryos, we needed to reduce the blastoderm shear viscosity by a factor ∼7 compared with WT embryos, consistent with the expectation that intercellular friction is strongly reduced after removal of ∼60% of deep cells. Collectively, these results support the notion that autonomous reduction of blastoderm surface tension drives overall doming movements, while active contraction of the deep cell layer promotes homogeneous blastoderm thinning.

A key prediction of our model is that EVL cells autonomously expand during doming. In physical terms, this implies that (1) the force exerted at the contact line on the EVL is not increasing to drive EVL spreading, and (2) the EVL is not exposed to external forces outside the contact line which would drive its deformation. These assumptions imply that the total EVL surface tension, which depends on both contractile forces generated within the tissue as well as viscous stresses arising from tissue flow, is uniform and only slightly decreases during doming (Figure S3J and Methods S1). To test this prediction, we performed compression experiments of deep cell-depleted embryos during doming to determine the absolute values of blastoderm and yolk cell surface tensions by relating the compression force to their deformation (Figure S3B). We used deep cell-depleted embryos, since active and viscous stresses arising within the blastoderm of those embryos are strongly diminished (Figures S3D–S3G), and we assumed that their influence on blastoderm surface tension can be neglected. When measuring yolk cell surface tension, we found that this tension remained constant during doming with *T*_y_ = 236.22 ± 41.31 pN/μm (n = 7) (Figure S3B), confirming our theoretical assumptions. When measuring the blastoderm surface tension, we found this tension to be close to the measured yolk cell tension (Figure S3B), in line with our theoretical predictions. Directly matching the measured value with the predicted value of blastoderm surface tension, however, was impossible due to high fluctuations in blastoderm shape changes during compression, leading to high variations in the measured blastoderm surface tensions between the different experiments (Figure S3B). Together, these observations support our model of EVL cells autonomously expanding during doming.

### Reduction in Blastoderm Surface Tension Is Required and Sufficient to Trigger Doming

**Figure 5:**
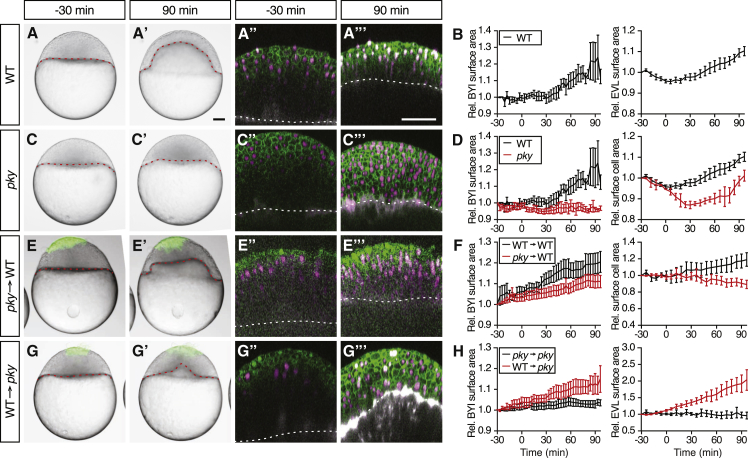
Reduction in Blastoderm Surface Tension Is Required and Sufficient to Trigger Doming (A–H) Intact WT (n = 6 embryos) (A) and (B) and *pky* embryos (n = 3 embryos) (C) and (D) and embryos where EVL/surface cells were transplanted from *pky* to WT embryos (E) and (F) (n = 10 embryos) and from WT to *pky* embryos (G) and (H) (n = 9 embryos). Bright-field images of mosaic embryos before (−30 min) and after completion of doming (+90 min) with transplanted cells marked by fluorescent dextran; green (A), (A′), (C), (C′), (E), (E′), (G), and (G′). Confocal images with plasma membrane expressing mem-GFP (green), nuclei marked by H2A-mCherry (magenta) and BYI outlined by fluorescent dextran (white); (A″), (A‴), (C″), (C‴), (E″), (E‴), (G″), and (G‴). Transplanted cells were marked by fluorescent dextran; green (E), (E′), (G), and (G′), or H2B-GFP; green (E″), (E‴), (G″), and (G‴). Red and white dashed lines, BYI. Changes in relative BYI area (left column) and relative EVL/surface cell area (middle column) as a function of time during doming in the different transplantation experiments (B), (D), (F), and (H). Note that the results from control experiments (WT into WT [n = 3 embryos] and *pky* into *pky* [n = 3 embryos]) were included in the plots as reference (F) and (H), see also Figures S5D–S5G, and that changes in relative EVL area in the transplantation experiments were determined exclusively in the region of the blastoderm where the transplanted cells were located. The WT data in (B) and (D) is the same as Figures 1G and 1J. Error bars, ±SEM. Scale bars, 100 μm. See also Figures S5 and S6; Movie S5.

We next sought to change EVL surface tension and determine how this affects doming. First, we asked whether EVL surface tension reduction is required for doming. To this end, we sought to inhibit doming in WT embryos by interfering with surface cell expansion. As a potential source of expansion-defective surface cells, we turned to maternal zygotic *poky* (*pky*) embryos, a mutant in the *conserved helix-loop-helix ubiquitous kinase* (*chuk*)/*IκB kinase 1* (*ikk1*) gene, where failed EVL differentiation has been associated with defective doming (Figures 5A–5D) (Correa et al., 2005, Fukazawa et al., 2010). To determine whether in *pky* mutants defective EVL differentiation leads to impaired surface cell expansion, we generated deep cell-depleted *pky* mutant embryos and analyzed surface cell expansion in mutant embryos lacking a large part of their deep cell layer. We found that surface cells were not expanding in deep cell-depleted *pky* embryos (Figure S5A), indicating that autonomous surface cell expansion is defective in the mutant. We then transplanted a patch of ∼70 expansion-defective surface cells from *pky* donor embryos onto the surface of WT host embryos, from which a similar-sized patch of EVL cells had been removed before the transplantation, and analyzed how doming movements in the host embryo are affected by the patch of expansion-defective *pky* surface cells (Figures 5E and S6A–S6C). Strikingly, we found that upward bulging of the BYI below the patch of transplanted cells was diminished and that movement of the contact line toward the vegetal pole slowed down (Figures 5F and S5B; Movie S5). This suggests that active surface cell expansion is required for doming.

Next, we asked whether active surface cell expansion is also sufficient to trigger doming. To this end, we sought to rescue doming in doming-deficient *pky* mutant embryos by transplanting WT donor EVL cells onto the surface of *pky* host embryos. We reasoned that locally rescuing surface cell expansion in *pky* mutant embryos might normalize their doming phenotype. Remarkably, we found that, in *pky* embryos on which WT surface cells were transplanted, the BYI bulged upward directly below the transplanted patch of donor surface cells undergoing expansion, indicative of a locally restricted rescue of doming (Figures 5G and 5H; Movie S5). This suggests that surface cell expansion is sufficient to trigger doming movements.

### Changes in Blastoderm Surface Tension Modulate Radial Stress within the Blastoderm

**Figure 6:**
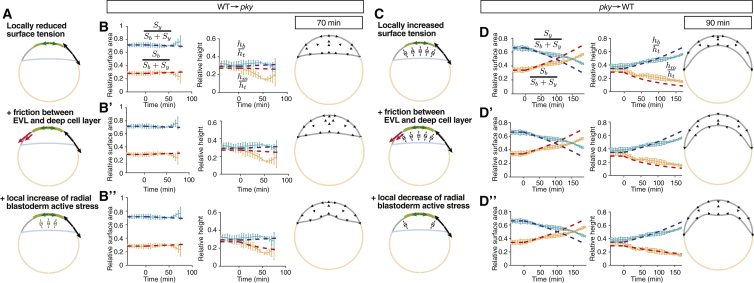
Simulations of the EVL/Surface Cell Transplantation Experiments (A) Schematic of possible mechanisms involved in rescuing doming by transplanting WT EVL cells on *pky* mutants: (1) reduced surface tension of the transplanted WT EVL cells reduces blastoderm surface tension; (2) in addition to reduced surface tension, a friction force between transplanted EVL cells and adjacent deep cell layer is included; and (3) in addition to reduced surface tension and friction, radial contraction of the deep cell layer below the grafted EVL is locally increased. (C) Corresponding mechanisms involved in *pky* mutant EVL transplants inhibiting doming in WT embryos: (1) increased surface tension in the grafted patch increases blastoderm surface tension, (2) in addition to increased surface tension, a friction force between EVL and deep cells is included, and (3) in addition to increase surface tension and friction, radial deep cell layer contraction below the grafted EVL is locally decreased. (B–B″ and D–D″) Simulation of changes in either *pky* embryo shapes as a result of WT EVL transplantations (B) and (B″) or WT embryo shapes as a result of *pky* surface cell transplantations (D) and (D″), according to the three mechanisms described in (A) and (C). Left plots display embryo surface area and height with pale blue and red curves showing the experimental measurements, and dashed red and blue thick lines the simulation results. Right panels show simulated embryo shapes with black arrows outlining the blastoderm velocity field. Error bars, ±SD. See also Figures S5 and Movie S6.

Our observations from the transplantation experiments between WT and *pky* mutant embryos suggest that local changes in blastoderm surface tension elicit local changes in upward bulging of the BYI. To understand how this can be explained by our mechanical description of the embryo, we first asked to what degree surface cell expansion and/or radial cell intercalations are defective in *pky* mutants. Our phenotypic analysis of *pky* embryos revealed that not only surface cells fail to expand (Figure S5C), but that also active radial contraction of the deep cell layer is defective (Figure S5C). Specifically, we found that, in *pky* mutants, deep cells showed reduced motility and did not elongate along the radial axis of the blastoderm (Figures S5D and S5E). We then asked whether simulations of doming based on both absence of active radial stress within the blastoderm and increased blastoderm surface tension give rise to embryo shape changes similar to the situation in *pky* mutants (Figure S5G). We found that such simulations closely phenocopied the *pky* mutant (Figure S5G), supporting the notion that both blastoderm surface tension reduction and radial contraction of the deep cell layer are defective in *pky* mutants. With the mechanical description of both *pky* and WT embryos in hand, we then performed simulations where we either locally reduced surface tension in a circular region around the animal pole in *pky* mutants or increased this tension in WT embryos, reflecting the transplantation of WT cells into *pky* and vice versa (Figures 6A and 6C). We found that this led to a local increase or reduction in surface cell expansion within the transplanted region, respectively, consistent with our experimental observations (Figures S5E–S5K″). However, we failed to reproduce the observed local changes in BYI upward bulging (Figures 6B and 6D). We then tested whether introducing friction force acting between surface cells and deep cells could trigger those local changes of BYI upward bulging observed in the transplantation experiments. We found, however, that including such friction force in our simulations did not lead to changes in BYI upward bulging of a magnitude observed in the experiments (Figures 6B′ and 6D′). This argues against such friction force playing a decisive role in mediating the effect of surface cell expansion on BYI upward bulging. Finally, we hypothesized that surface cell expansion not only reduces surface tension, but also locally increases radial stress within the blastoderm. Strikingly, introducing such an effect in our simulations led to changes in embryo geometry closely matching the experimental observations in our transplantation experiments (Figures 6B″ and 6D″; Movie S6). This suggests that active surface cell expansion functions in doming by both reducing blastoderm surface tension and promoting radial stress within the blastoderm.

To test the prediction from our model that surface cell expansion triggers radial deep cell layer contraction, we first asked whether in our transplantation experiments surface cell expansion indeed locally triggers radial stress generation within the underlying deep cell layer. To this end, we analyzed deformation of yolk granules in *pky* mutant embryos where a patch of transplanted WT EVL cells induced local upward bulging of the BYI (Figures 5G and 5H; Movie S5). We found that the yolk granules were locally elongated along the radial axis at the position where the BYI bulged upward (Figure S5F). This supports the assumption that local stresses generated within the deep cell layer are transmitted to the yolk cell, locally deforming yolk granules.

**Figure 7:**
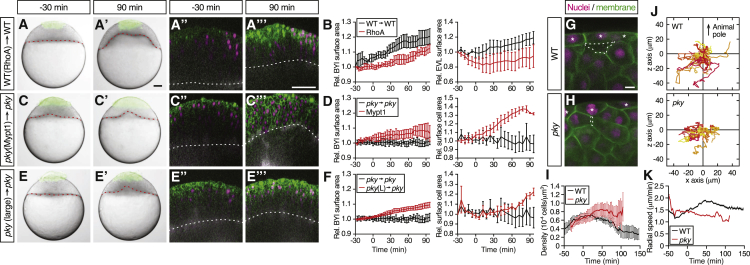
Surface Cell Expansion Reduces Deep Cell Density and Increases Deep Cell Motility (A–F) Transplantation of EVL/surface cells from RhoA-overexpressing WT to WT embryos (A) and (B) (n = 3 embryos), Mypt1-overexpressing *pky* to *pky* embryos (C) and (D) (n = 3 embryos), and of large patches of *pky* surface cells replacing a smaller patch of surface cells in *pky* embryos (E) and (F) (n = 3 embryos). Bright-field images of mosaic embryos before (−30 min) and after completion of doming (+90 min) with transplanted cells marked by fluorescent dextran; green (A), (A′), (C), (C′), (E), and (E′). Confocal images with plasma membrane expressing mem-GFP (green), nuclei marked by H2A-mCherry (magenta) and BYI outlined by fluorescent dextran (white); (A″), (A‴), (C″), (C‴), (E″), and (E‴). Transplanted cells were marked by fluorescent dextran; green (A), (A′), (C), (C′), (E), and (E′), or H2B-GFP; green (A″), (A‴), (C″), (C‴), (E″), and (E‴). Red and white dashed lines, BYI. Changes in relative BYI area (left column) and relative EVL/surface cell area (right column) as a function of time during doming in the different transplantation experiments (B), (D), and (F). The control data in (B), (D), and (F) were taken from Figures 5F and 5H. Error bars, ±SEM. Scale bars, 100 μm. (G–I) Exemplary confocal images of deep cell clustering below EVL/surface cells at the onset of doming in WT (G) and *pky* embryos (H). Space between deep cells is marked by a dashed line. Asterisks, EVL/surface cells. Plasma membrane was labeled by mem-GFP (green) and nuclei by H2A-mCherry (magenta). Density of deep cell nuclei in WT and *pky* mutant embryos was quantified within an area of ∼50 μm below EVL/surface cells as a function of time during doming (I). Scale bars, 10 μm. Error bars, ± SEM, n = 6 embryos for WT and *pky* each. (J and K) Exemplary trajectories of deep cell movements beneath EVL/surface cells. Nuclei of deep cells within and area of ∼60 μm below EVL/surface cells were tracked in WT (top, n = 57 cells) and *pky* embryos (bottom, n = 35 cells) for 100 min from the onset of doming and plotted from the origin (J). The z axis corresponds to the animal-vegetal axis of the embryo with animal pole up. Radial speed of deep cells in WT and *pky* mutant embryos within an area of ∼50 μm below EVL/surface cells (K). Error bars, ±SEM, n = 6 embryos for WT and *pky* each. See also Movie S5.

Next, we asked whether the ability of surface cells to expand, rather than any other features of the EVL that might be different between WT and *pky* mutant embryos, is responsible for the observed effect on deep cell layer contraction in our transplantation experiments. To this end, we transplanted EVL cells from WT embryos overexpressing the small GTPase RhoA onto WT embryos (Figure 7A). We reasoned that overexpression of RhoA in the donor EVL cells will promote actomyosin contraction in those cells (Tinevez et al., 2009) and thus reduce their ability to expand on the blastoderm surface. We found that RhoA-overexpressing EVL cells transplanted onto the surface of WT host embryos failed to expand (Figure 7B), and that this failure was accompanied by reduced doming movements in the host embryo (Figure 7B and Movie S5). Notably, RhoA-overexpressing EVL cells not only failed to actively expand but also showed a tendency to delaminate into the deep cell layer at the end of doming (90 min; Figure S6H). However, this delamination typically occurred after the effect on doming movements became visible in the transplanted embryos (not shown), arguing against this function of RhoA being responsible for the observed doming defect. Conversely, we also performed transplantation of either a large patch of *pky* donor surface cells (∼100 cells) replacing a smaller patch of surface cells (∼60 cells) in *pky* host embryos or, alternatively, of a patch of *pky* donor surface cells overexpressing *myosin phosphatase 1* (*mypt1*) (Jayashankar et al., 2013), which reduces actomyosin contraction in those cells, replacing a similar-sized patch of surface cells in *pky* hosts (Figures 7C and 7E). We reasoned that in both of these transplantations, we locally normalize expansion of *pky* surface cells without changing their genotype (Figure S6I), allowing us to specifically analyze the effect of modulating surface cell expansion on doming in *pky* mutants. Strikingly, we found that in both of these transplantation assays, the area of transplanted surface cells expanded and the BYI below the transplanted surface cells displayed considerable upward bulging, indicative of a local rescue of doming in *pky* (Figures 7C–7F). Together, these transplantation experiments confirmed our assumption that expansion of surface cells, rather than other not yet identified activities of surface cells, triggers radial deep cell layer contraction.

Finally, we asked by which mechanism surface cell expansion might trigger radial deep cell layer contraction. Assuming that deep cell-cell contact formation has previously been shown to reduce cell protrusion formation and movement (Arboleda-Estudillo et al., 2010), we reasoned that surface cell expansion might promote radial deep cell layer contraction by locally reducing cell density at the surface of the underlying deep cell layer allowing deep cells to become motile and actively undergo radial cell intercalations. To test this hypothesis, we analyzed changes in deep cell density and motility at the deep cell layer surface as a function of surface cell expansion at the onset of doming. We found that in WT embryos, deep cell density at the deep cell layer surface decreased and deep cell motility increased when surface EVL cells started to expand at the onset of doming (Figures 7G and 7I). This anti-correlation between deep cell density and radial deep cell motility was particularly evident during the first 50 min of doming, while at later stages of doming radial deep cell motility begun to decrease despite a continuous decrease in cell density (Figures 7G and 7I). In contrast to the situation in WT embryos, no such anti-correlation between deep cell density and motility during early doming were observed when surface cells fail to expand in *pky* mutant embryos (Figures 7H–7K). These observations support our assumption that during early stages of doming surface cell expansion promotes radial deep cell layer contraction by locally providing sufficient space required for active deep cell intercalation.

## Discussion

Doming is achieved by the coordinated expansion and thinning of the blastoderm. Our study shows that the EVL, by reducing its surface tension, simultaneously drives both of these processes thereby coordinating them. The EVL exerts its dual function through two interdependent mechanisms: (1) by reducing its surface tension the EVL generates an imbalance of forces acting on the contact line between EVL, yolk cell, and the BYI, driving contact line motion toward the vegetal pole and thus blastoderm expansion. Assuming that the relative volume of the yolk is conserved, the reduction in EVL surface tension is expected not only to drive blastoderm expansion, but also to change the balance of yolk to blastoderm pressure at the BYI, causing the BYI to bulge upward and thus thin the blastoderm. (2) By expanding, the EVL also induces active radial cell intercalation within the underlying deep cell layer, generating radial stress within the blastoderm required for homogeneous blastoderm thinning during doming.

Expansion of epithelial surface cells in different fish species has previously been associated with both active spreading (Betchaku and Trinkaus, 1978) and passive stretching due to forces generated by an actomyosin cable within the yolk syncytial layer (YSL) pulling on the margin of the epithelium (Behrndt et al., 2012). Our observations suggest that surface cells autonomously reduce their surface tension, thereby undergoing spreading, and that this process depends on EVL cell fate specification. This does not exclude a cell-non-autonomous component in EVL spreading, such as pulling forces from the YSL, but points at an important role of EVL cell differentiation for the spreading process itself. How EVL cell differentiation results in EVL cell spreading is unknown. Given that EVL differentiation is accompanied by a pronounced upregulation of keratin expression (Imboden et al., 1997), and that keratins have previously been implicated in cell spreading during wound healing (Bragulla and Homberger, 2009), it is conceivable that the build-up of a keratin-cytoskeleton is involved in EVL cell spreading.

The molecular and cellular mechanisms by which deep cells undergo radial cell intercalations are not yet fully understood (Lepage and Bruce, 2010). E-cadherin-mediated preferred attachment of deep cells to the overlying EVL cells has been proposed to constitute one potential mechanism driving deep cell radial cell intercalation (Kane et al., 2005, Shimizu et al., 2005). Consistent with such a role of EVL in driving radial cell intercalations are our observations that EVL expansion both reduces deep cell density and increases deep cell motility (Figures 7G–7K). It is thus conceivable that EVL expansion triggers radial deep cell intercalations by expanding the substrate to which deep cells preferably anchor and providing sufficient space for deep cells reaching this substrate. In addition, or alternatively, the complement factor C3 has recently been involved in radial deep cell intercalation during *Xenopus* gastrulation (Szabó et al., 2016). Specifically, it has been proposed that a short-range gradient of C3 originating from surface cells attracts deep cells to move toward the surface, resulting in radial deep cell intercalations. While our preliminary observations using drugs blocking C3 signaling do not support a critical role of C3 in doming (data not shown), our data are in principle compatible with such a function of C3 also in zebrafish gastrulation. However, chemoattraction by C3 released from surface cells is unlikely to explain why surface cell expansion triggers active radial deep cell intercalation, unless one assumes that surface cell expansion relates to the amount of C3 released by these cells. Whether such elaborate relationship exists and to what extent it would be needed to explain the effect of surface cell expansion on radial deep cell intercalations remains to be investigated.

For developing a mechanical model of the doming process incorporating the effects of both surface cell expansion and deep cell intercalations, we have proposed an active fluid description of embryo-scale deformation. In this description, force generation in surface and deep cells of the embryo is characterized on a scale much larger than a cell by a few phenomenological parameters corresponding to tissue viscosities and internal force generation (Etournay et al., 2015, Ranft et al., 2010). We have specifically investigated here the mechanical interplay between two tissue types, an epithelial surface layer and mesenchymal deep cells positioned below this layer, undergoing pronounced shape changes during doming. Despite its simplicity, our physical description allowed us to capture the characteristic shape changes of those tissues during doming (Figures 4D and 4F). Furthermore, we were able to measure embryo surface tension by embryo compression experiments, blastoderm viscosity by explant fusion experiments, and obtain physical values of other internal viscosities and stresses by adjusting the parameters of our physical description to match the tissue shape changes occurring during doming. Interestingly, we found that the value of blastoderm viscosity is ∼10–100 times smaller than reported for other tissues (Forgacs et al., 1998, Guevorkian et al., 2010, Marmottant et al., 2009), possibly due to the high motility of deep cells in the blastoderm reducing effective tissue viscosity. Moreover, the internal anisotropic stresses generated within the blastoderm turned out to be in a magnitude of ∼0.5 Pa (Table S1), smaller than typically exerted by, e.g., actomyosin networks (Mathur et al., 2001). This suggests that comparably small tissue stress anisotropies acting over ∼1 hr of doming are sufficient to trigger pronounced embryo shape changes during this process.

The coordinated spreading of multiple tissues constitutes a universal mechanism by which embryos take shape during gastrulation (Solnica-Krezel, 2005). Thus, elucidating the force-generating processes by which tissues undergo coordinated spreading is central for understanding the physical basis of embryo morphogenesis. Our findings provide evidence that for spreading of complex multilayered tissues consisting of both epithelial surface cells and mesenchymal deep cells, surface cells play a key role. By reducing their surface tension and thus actively expanding, they not only drive tissue expansion, but also trigger active radial intercalation of underlying deep cells, a process required for homogeneous tissue thinning during spreading. Thus, by simultaneously reducing tissue surface tension and increasing radial tissue contraction, active surface cell expansion allows complex tissues to undergo coordinated tissue expansion and thinning.

## STAR★Methods

### Key Resources Table

**Table:** 

Reagent or Resource	Source	Identifier
**Antibodies**		

anti-phospho-Histone H3 (Ser 10)	Upstate	Cat#06-570
anti-ZO1	Invitrogen	Cat#33-9100
Cy5-conjugated goat anti-mouse IgG	Jackson ImmunoResearch	Cat#115-175-146

**Chemicals, Peptides, and Recombinant Proteins**		

DAPI	Invitrogen	Cat#D1306
Hydroxyurea	Sigma-Aldrich	Cat#H8627
Aphidicolin	Sigma-Aldrich	Cat#A0781
Dextran Alexa Fluor 647	Invitrogen	Cat#D22914
Dextran fluorescein	Invitrogen	Cat#D1821
Histone H1 Alexa Fluor 647	Invitrogen	Cat#C29926

**Critical Commercial Assays**		

mMESSAGE mMACHINE SP6 Transcription Kit	Ambion	Cat#AM1340

**Experimental Models: Organisms/Strains**		

Zebrafish: *AB*	Zebrafish International Resource Center (ZIRC)	ZFIN: ZDB-GENO-960809-7
Zebrafish: *chuk*^*p20ad/p20ad*^ (*poky*)	Fukazawa et al., 2010	ZFIN: ZDB-FISH-150901-16711
Zebrafish: *Tg(actb1:lifeact-EGFP)*	Behrndt et al., 2012	N/A
Zebrafish: *Tg(actb1:myl12.1-EGFP)*	Maître et al., 2012	N/A
Zebrafish: *Tg(krt4:EGFP-CAAX)*	Choo et al., 2006	ZFIN: ZDB-ALT-111207-5

**Recombinant DNA**		

Plasmid: Membrane-GFP	Kimmel and Meyer., 2010	N/A
Plasmid: Membrane-RFP	Iioka et al., 2004	N/A
Plasmid: H2B-EGFP	Keller et al., 2008	N/A
Plasmid: H2A-mCherry	Arboleda-Estudillo et al., 2010	N/A
Plasmid: RhoA	Takesono et al., 2012	N/A
Plasmid: Mypt1	Jayashankar et al., 2013	N/A

**Software and Algorithms**		

Mathematica	Wolfram Research, Inc.	http://www.wolfram.com
Fiji	NIH	https://fiji.sc
Matlab2013a	MathWorks Inc.	http://mathworks.com
Octave 4.0.0	GNU Octave	http://www.octave.org
Imaris 7.4	Bitplane Inc.	http://www.bitplane.com
Packing Analyzer	Aigouy et al., 2010	N/A

### Contact for Reagent and Resource Sharing

For further information, requests should be directed to and will be fulfilled by the Lead Contact, Carl-Philipp Heisenberg (heisenberg@ist.ac.at).

### Experimental Model and Subject Details

#### Zebrafish (*Danio rerio*)

Zebrafish strains were maintained under a 14-hr light/10-hr dark cycle (Westerfield, 2007). All experiments using zebrafish in this study were approved by the responsible Austrian legal authorities. The following zebrafish strains were used in this study: wild type (WT) AB, *poky* (*pky*) (Wagner et al., 2004), *Tg(actb1:lifeact-EGFP)* (Behrndt et al., 2012), *Tg(actb1:myl12.1-EGFP)* (Maître et al., 2012) and *Tg(krt4:EGFP-CAAX)* (Choo et al., 2006).

### Methods Details

#### Handling of Zebrafish Embryos

Zebrafish embryos were kept in E3 embryo medium and staged as previously described (Kimmel and Law, 1985). Embryonic manipulations were done in Danieau's solution [58 mM NaCl, 0.7 mM KCl, 0.4 mM MgSO_4_, 0.6 mM Ca(NO_3_)_2_, 5 mM HEPES (pH 7.2)] unless otherwise stated.

#### DNA Constructs and mRNA/Dye Injections

The following expression constructs were used: membrane-GFP (mem-GFP) (Kimmel and Meyer, 2010), membrane-RFP (mem-RFP) (Iioka et al., 2004), H2B-EGFP (Keller et al., 2008), H2A-mCherry (Arboleda-Estudillo et al., 2010), RhoA (Takesono et al., 2012), and N-terminal fragment of Mypt1 (Jayashankar et al., 2013). mRNA was synthesized using mMESSAGE mMACHINE SP6 kit (Ambion). Zebrafish embryos were injected using glass capillary needles (30-0020, Harvard Apparatus, MA, USA), which were pulled by a needle puller (P-97, Sutter Instrument) and attached to a microinjector system (PV820, World Precision Instruments). 200 pg *mem-GFP*/*mem-RFP*/*H2B-EGFP*/*H2A-mCherry*, 50 pg *rhoA*, and 100 pg *mypt1* mRNA were injected into 1-cell stage embryos. To label the BYI, a cocktail of 2 mg/mL dextran Alexa Fluor 647 (10,000 MW; D22914, Invitrogen) and 2 mg/mL histone H1 Alexa Fluor 647 (C29926, Invitrogen) diluted in 50% glycerol was injected into the yolk of high-oblong stage (3.3–3.7 hpf) embryos.

#### Image Acquisition

For microscopy, dechorionated embryos were mounted in 0.3% low melting point (LMP) agarose (16,520-050, Invitrogen) either on a mold made by 1% LMP agarose for upright microscopy or on a glass bottom dish (P35G-1.5-14-C, MatTek Corporation) for inverted microscopy. Mounted embryos were kept in an incubation chamber at 28.5°C during microscopy. For bright-field microscopy of whole embryos, embryos were imaged on a Nikon Eclipse inverted widefield microscope equipped with CFI Plan Fluor 10x/0.3 objective (Nikon), a fluorescent light source (Lumencor) and a band-pass filter 438/24 for observing dextran fluorescein in a 1.25 × 0.94 mm area as 640 × 480 pixels with 1 min time intervals. For laser scanning microscopy of deep tissues, embryos were imaged on a TriM Scope two-photon microscope (LaVision BioTec), which was equipped with a Chameleon Ultra II laser with Chameleon Compact OPO (Coherent), a Plan-Apochromat 20x/1.0 water-immersion objective (Zeiss) and GaAsP detectors (Hamamatsu Photonics), in a 400 × 400 μm area as 341 × 341 pixels in about 300 μm depth with 3 μm step size. Images were obtained every 3 min with excitation wavelengths of 800 nm (Ti-sapphire laser) and 1,100 nm (OPO). The fluorescent signal was collected using bandpass filters of 525/50 nm (GFP), 629/56 nm (mCherry, RFP) and 675/67 nm (Alexa 647) split by longpass filters of 593 nm and 650 nm. For bright-field and confocal imaging of more superficial tissues and fixed specimen, embryos were imaged on a Leica SP5 upright microscope equipped with a HCX IRAPO L 25x/0.95 water-immersion objective (Leica), an argon laser (488 nm) and a HeNe laser (561 and 633 nm), or on a Leica SP5 inverted microscope equipped with an HC PL APO 20x/0.7 objective (Leica), an argon laser (488 nm) and a HeNe laser (561 and 633 nm) in about 250 × 250 or 620 × 620 μm area as 512 × 512 or 1024 × 1024 pixels, respectively, with 1–3 μm step sizes depending on the objectives used.

#### Embryo Geometrical Parameter Analysis

To quantify embryo shape, side-view bright-field time-lapse images of the embryo were taken by using a Nikon Eclipse microscope (Nikon). Fiji (NIH) was then used to mark several points on EVL, BYI, and the interface of the yolk to the medium. To fit splines to the marked points, a set of custom GNU Octave (version 4.0.0, GNU Octave) scripts was used. From these splines, the interface area, bulk volume, and the other observables were determined by assuming that the embryo is rotationally symmetric around its animal-vegetal (AV) axis.

#### Embryo Compression and Relaxation Experiments

Dechorionated embryos were placed on the lower glass plate within the incubation chamber of a MicroSquisher (CellScale) filled with Danieau's solution. The embryo was oriented within the chamber using the upper glass plate, which was attached by glue to a tungsten beam with 0.076 mm diameter, 400 GPa modulus and 60–63 mm length (CellScale). The embryo was then compressed with the upper plate by 20% of its initial uncompressed height, starting from oblong-sphere stage (3.7–4 hpf). Lateral-side views of embryo morphology were recorded for up to 150 min after the onset of compression. The contact areas and contact angles between the embryo and the glass plates and the curvature of the embryo were measured from the images of the compressed embryos using custom-built scripts in Fiji and Matlab (version 2013a, MathWorks). To avoid using ambiguous interfaces between embryo and plates for the measurements, the contact area/angle were measured about 25 μm away from the plates toward the inside. These geometrical parameters together with the recorded compression force were used to calculate the surface tensions of the embryo (see Methods S1). To measure the viscosity of the blastoderm, tissue explants were excised from the blastoderm of WT embryos at high stage (3 hpf) using forceps in Danieau's solution. Two blastoderm explants were then fused at their wounding site and cultured for at least 1.5 hr in the same medium at room temperature. When unperturbed control embryos had reached the onset of doming (4 hpf), the fused explants were compressed using a MicroSquisher. Explant surface tension was calculated as described above. Explant fusion was recorded using a Nikon Eclipse inverted wide-field microscope equipped with CFI Plan Fluor 10x/0.3 objective (Nikon) in a 1.25 × 0.94 mm area as 640 × 480 pixels with 1 min time intervals. To measure the long axis of the explants, the explant outline was fitted with an ellipse using the ‘Fit Ellipse’ function of Fiji, and the major axis of the fitted ellipse was taken as the longest axis of the explant. For the compression-relaxation experiment of yolk explants, the blastoderm of WT embryos between 256- and 512-cell stages (2.5–2.75 hpf) was removed from the yolk cell using forceps in Danieau's solution. The isolated yolk explants were then placed in the same medium as unperturbed control embryos until the control embryos reached the onset of doming (4 hpf) and compressed by 20% of their initial height using a MicroSquisher. After 30 min of constant compression the upper plate was removed and the recovery of the explant height was recorded and manually measured using a custom-built Fiji script.

#### Immunostaining

Embryos were fixed in 4% paraformaldehyde (PFA) for 1.5 hr at room temperature or overnight at 4°C. After fixation, they were washed with PBS containing 0.1% Tween 20 (PBST) and dechorionated in PBST. Washed embryos were subsequently incubated in blocking solution (10% fetal calf serum in PBST) for 1 hr at room temperature, and then exposed to primary and secondary antibody solutions overnight at 4°C. As primary antibody, rabbit anti-phospho-histone H3 (1:200; 06–570, Upstate) and mouse anti-ZO1 (1:100; 33–9100, Invitrogen) were used. As secondary antibody, Cy5-conjugated goat anti-mouse IgG (1:500; Jackson ImmunoResearch) was used. To label nuclei, 50 μg/mL DAPI was added to the secondary antibody solution.

#### Cell Division Inhibition

Dechorionated embryos were treated with a cocktail of 60 mM hydroxyurea (H8627, Sigma) and 300 μM aphidicolin (A0781, Sigma) (HUA) from high-oblong stage (3.3–3.7 hpf) and kept in this solution throughout the doming period. The efficiency of cell division inhibition was examined by calculating the ratio of pHH3-positive nuclei and total nuclei number.

#### Deep Cell Removal and Replacement

Deep cells were aspirated from dechorionated embryos at 1k-oblong stage (3–3.7 hpf) using a glass pipette with an inner diameter of 45 μm and a spiky tip (BioMedical Instruments, Germany) connected to a syringe for manually controlling the suction pressure. The suction pipette was inserted into the blastoderm at a maximum of three different positions close to the EVL margin to avoid any unspecific interference with EVL epiboly movements. DEL cell-depleted embryos were cultured for at least 20 min before being injected with Danieau's solution. Danieau's solution was injected into the embryos until the volume of blastoderm became similar to the blastoderm before DEL cell removal.

#### EVL/Surface Cell Transplantation

Transplantation of EVL/surface cells was performed as described before (Morita et al., 2012) with the following modifications: the host and donor embryos were injected with *mem-GFP*/*mem-RFP*/*H2B-EGFP*/*H2A-mCherry* mRNA or 1 mg/mL dextran fluorescein (10,000 MW; D1821, Invitrogen) at the 1-cell stage, dechorionated at high-oblong stage (3.3–3.7 hpf) and then transferred to Ca^2+^-free Ringer's solution [116 mM NaCl, 2.9 mM KCl, 5 mM HEPES (pH 7.2)]. A part of the blastoderm (EVL and deep cells) was removed from the donor embryo using forceps, and deep cells attaching to this blastoderm explant were shaved off by an eyebrow that was fixed at the tip of a pasteur pipette in order to isolate a single layer of EVL/surface cells. Before transplantation, EVL/surface cells were excised from the host embryo using forceps with minimal removal of adjacent deep cells. The patch of donor EVL/surface cells was then placed on the EVL-surface cell-depleted site of the host embryo. Transplanted embryos were cultured for at least 30 min at 25-28.5°C. The number of co-transplanted deep donor cells constituted on average no more than 3.2% ± 0.36 (SEM) of the untransplanted host deep cells.

#### Theoretical and Computational Models

Theoretical and computational models used in this study are described in Methods S1.

### Quantification and Statistical Analysis

#### Image Analyses

Laser scanning two-photon microscopy images were processed using Fiji (NIH) as follows: images were converted from original 16-bit type to 8-bit type at a certain threshold level. Because the red (mCherry and RFP) and far-red (Alexa 647) fluorescence slightly bled through into the other channel's detector (i.e. red fluorescence into the far-red detector and vice versa), images from these channels were subtracted from each other between the same *x*, *y* and *z* pixel positions based on the signal intensities. Images were subsequently processed by image registration using the Fiji plugin Correct 3D drift (Parslow et al., 2014) and then used for further analyses. To average results from different embryos, embryo images were temporally aligned by using the beginning of the last nuclei division within external yolk syncytial layer (YSL), which is a known characteristic feature of embryos at the onset of doming (Kimmel and Law, 1985). For BYI surface area (3D) measurements, dextran signals of BYI were processed by filtering using Gaussian blur and binarization, and detected at their closest position to the interface with the blastoderm using a custom-built Fiji script. The BYI surface area was then calculated by connecting those detected dextran signal points as a surface using Matlab. Deep cell radial speed was measured by processing two-photon microscopy-obtained images of blastoderm nuclei using the Spots Object function in Imaris (version 7.4, Bitplane), which allows to track the movement of each nucleus over time and obtain *x*-, *y*- and *z*-coordinates of their movements. To measure the radial speed of deep cells from the tracking data, the three-dimensional prospective center of the embryo was first determined at each time point (Figures S7A and S7B). To this end, embryo side view image stacks were made by the Fiji ‘Reslice’ function along *x* and *y* axes, respectively (Figure S7A). These image stacks were processed with maximum intensity projection and then binarized with a threshold in gray scale value, which includes about 30% darker pixels of total number of pixels. The arc contours of the binary images corresponding to the embryo outer surface were detected and fitted with the Fiji ‘Fit Circle’ function (Figure S7B). These circles were then used to determine the *x*-, *y*- and (mean) *z*-coordinates of the embryo center. To obtain the radial speed, the distance between each tracked nucleus and the embryo center was used to calculate the radial displacement of deep cells using Matlab. In embryos containing transplanted EVL/surface cells, the deep cell speed was measured only under the transplanted cells. For EVL surface area measurements, two-photon microscopy images of embryos expressing mem-GFP or mem-RFP were used. Because the images from two-photon microscopy contained signals from both the EVL/surface cells and the underlying deep cells, images were first processed by deleting signals from deep cells in each *z* slice after Gaussian filtering, binerization and determining the area which was created by reducing the outer contour of binerized image by the assumed thickness of the surface layer (Figures S7C–S7K). The processed images were assembled as a maximum z-projection, followed by semiautomatic segmentation using Packing Analyzer (v6.5; Aigouy et al., 2010), 3D correction of the segmented images and measurement of the surface area using a custom-built Matlab script. The surface area was measured for cells (and daughter cells after division) that remained within the image frame throughout the analyzed time points. To measure the fluorescent intensity of Lifeact-EGFP and Myl12.1-EGFP at the BYI, optical single z sections were obtained using two-photon microscopy in a ∼100 μm depth from the embryo surface. The BYI was manually spotted, and the mean gray values of the fluorescent signal were measured within a ∼100 μm region of the YSL below the BYI. The measured values were divided by the ratio of the mean gray values of the whole image at each time point over those at the initial time point (0 min) to correct for bleaching. For the aspect ratio and angles of the deep cells and yolk granules, mem-RFP and dextran Alexa 647 signals were used to detect their membrane and cytoplasm, respectively. The images were segmented with Packing Analyzer and then measured with a custom-built Fiji script that fits an ellipse to each cell/granule shape. To measure the angle of the cell's major axis, the center of the embryo section was determined by fitting a circle to the contour of the section image and the connected to the center of the fitted ellipse of the cell by a line. To determine the alignment of the cell's major axis with the radial axis of the embryo, the angle between this line and the cell's major axis was measured. To determine the preferred orientation of the yolk granules, the angle between the major axis of yolk granules and the horizontal line of the image was measured after tilting the whole image so that the BYI before doming was oriented parallel to the horizontal line of the image. To quantify the subcellular localization of actin in deep cells, the image was first segmented with Packing Analyzer using mem-RFP expression for outlining individual cells. The segmented area was then further subdivided into 12 sectors with the center of those sectors being aligned with the center of a fitted ellipse and also divided by a circumferential line which surrounds about 3 μm inside from the plasma membrane (Figure 3B, orange area), creating small compartments at the edge of each cell shape. Finally, the mean gray value of each small compartment was calculated and normalized by the value from 0-30 degree region from the major axis in order to localize signal intensity relative to the major axis of the fitted ellipse. To analyze the density and speed of deep cells near the surface of the embryo, the position of the outer surface of the embryo was first determined from confocal microscopy images of blastoderm cells expressing mem-GFP by using a custom-built Matlab script. In short, thresholded binary images of embryo side views were used to identify the outer surface of the blastoderm in a single pixel resolution, followed by treating the extracted surface with an averaging filter with 50 × 50 pixel size. The distance of deep cells from the embryo surface was determined by calculating the distance between each deep cell nucleus, which was detected by using the Spot Object function of Imaris, and its nearest point on the embryo surface. The density and radial speed of deep cells were measured in a region around the animal pole with an area of 100 × 100 μm in the equatorial plane and 50 μm away from the surface.

#### Statistical Analysis

Statistical details of experiments are reported in the figures and figure legends. Statistical significance between two groups was determined by two-tailed Student's *t*-test. Statistically significant differences are: ^∗^p < 0.05 and ^∗∗∗^p < 0.001.

## Author Contributions

H.M., G.S., and C.-P.H. designed the research. H.M. performed most of the experiments. H.M. and S.F.G.K. performed the compression experiment. H.M., S.G., M.B., S.F.G.K., and G.S. analyzed the experimental data. G.S. developed the theoretical model. S.G., M.B., and G.S. performed the simulations. H.M., G.S., and C.-P.H. wrote the manuscript.

## Supporting Citations

The following references appear in the Supplemental Information: Clift et al., 2005, De Gennes, 1985; Huh and Scriven, 1971, Moffatt, 1964, Zienkiewicz and Taylor, 1977.

## Supplemental Information

Document S1. Figures S1–S7 and Methods S1

Table S1. Values from Experimental Measurements and Lists of Parameters Used in the Simulations, Related to Figures 2, 4, 6, S2, S3, S4, and S5

Movie S1. Embryo Doming, Related to Figure 1

Movie S2. Simulations of Embryo Doming, Related to Figure 2

Movie S3. Deep Cell Movesment and Polarization during Doming, Related to Figure 3

Movie S4. Doming of Deep Cell-Depleted Embryo, Related to Figure 4

Movie S5. Doming in EVL/Surface Cell Transplanted Embryos, Related to Figures 5 and 7

Movie S6. Simulations of Doming in EVL/Surface Cell Transplanted Embryos, Related to Figure 6

Document S2. Article plus Supplemental Information
