# Supplementary material for: The Physical Basis of Coordinated Tissue Spreading in Zebrafish Gastrulation
Source: Dev Cell. 2017 Feb 27;40(4):354–366.e4. doi: 10.1016/j.devcel.2017.01.010 (PMC5364273; doi:10.1016/j.devcel.2017.01.010)
Supplement: Document S1. Figures S1–S7 and Methods S1 [file mmc1.pdf]

Developmental Cell, Volume *40*

Supplemental Information

The Physical Basis of Coordinated Tissue Spreading in Zebrafish Gastrulation

Hitoshi Morita, Silvia Grigolon, Martin Bock, S.F. Gabriel Krens, Guillaume Salbreux, and Carl-Philipp Heisenberg

Supplemental Inventory

Supplemental Figures

Figure S1: related to Figure 1

Figure S2: related to Figures 2 and 3

Figure S3: related to Figure 4

Figure S4: related to Figure 4

Figure S5: related to Figures 5 and 6

Figure S6: related to Figure 5

Figure S7: related to STAR Methods

Supplemental Tables

Table S1: related to Figures 2, 4, 6, S2, S3, S4 and S5

Supplemental Movies

Movie S1: related to Figure 1

Movie S2: related to Figure 2

Movie S3: related to Figure 3

Movie S4: related to Figure 4

Movie S5: related to Figures 5 and 7

Movie S6: related to Figure 6

Supplementary Theory

Methods S1: related to STAR Methods

Supplemental Figures

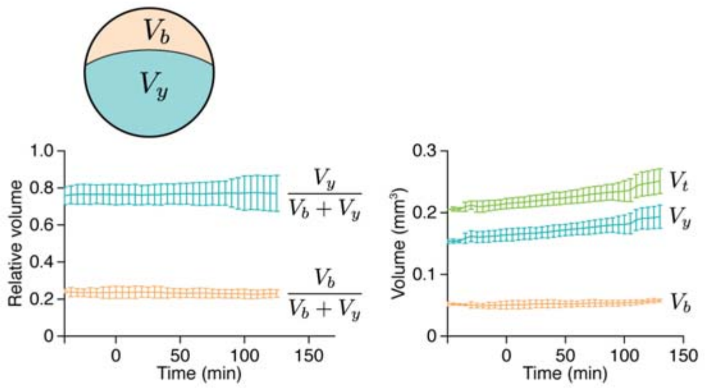

Figure S1. Changes in Embryo Volume during Doming, Related to Figure 1

Quantification of relative (left) and total (right) WT embryo volumes from bright-field embryo images. V_b, blastoderm volume; V_y, yolk volume; $V_t = V_b + V_y$, total volume. *n* embryos = 6. Error bars, ± s.d.

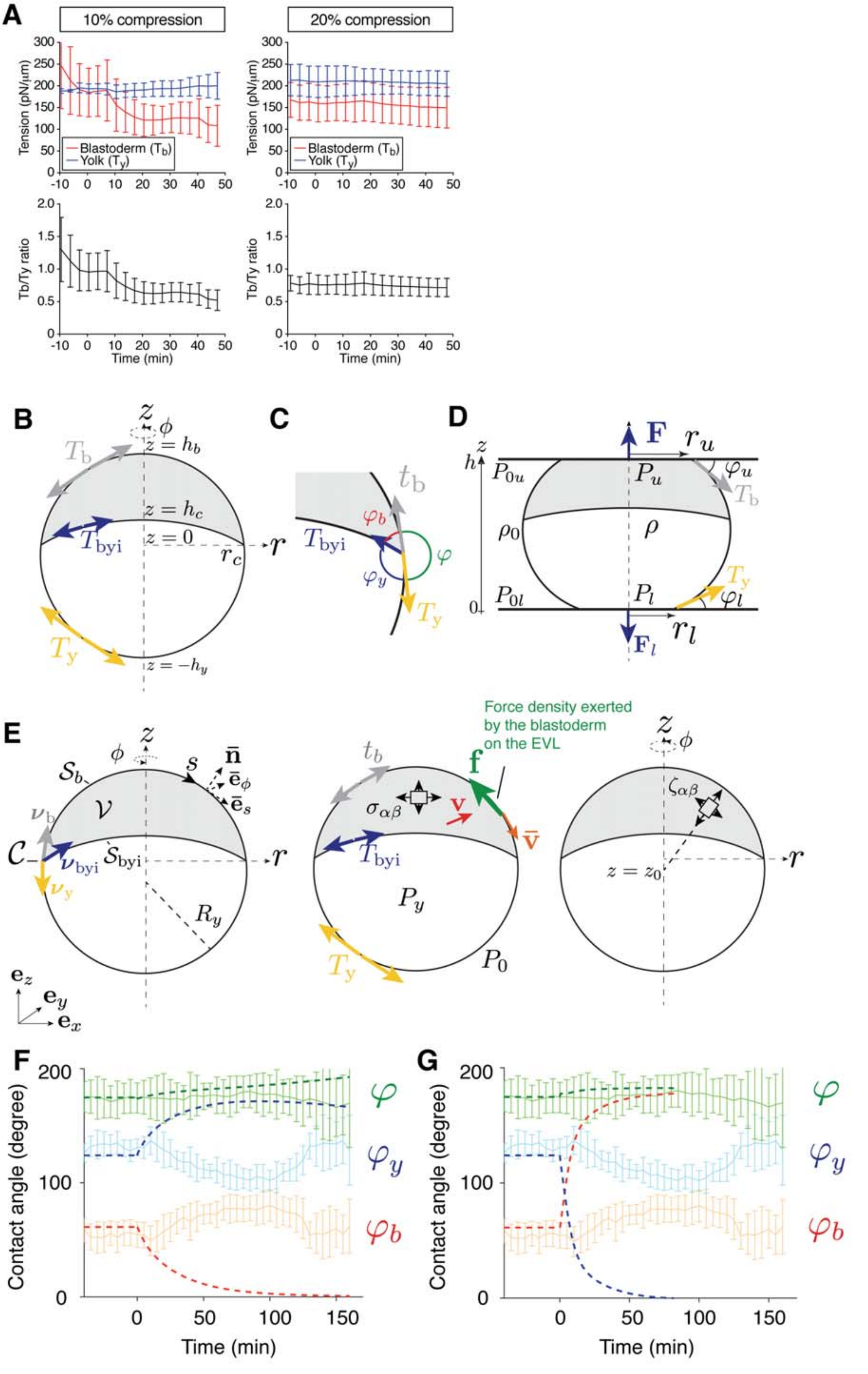

(legend on next page)

Figure S2. Physical Model of Doming, Related to Figures 2 and 3

(A) Quantification of T_b (red lines) and T_y (blue lines) and T_b/T_y (black lines) as a function of time after compression of the embryo by 10% (left panels) and 20% (right panels) of its initial uncompressed height during doming. *n* embryos (10%) = 5; *n* embryos (20 %) = 15. Error bars, ± s.d.

(B) Geometry and surface tension model of the zebrafish embryo. The blastoderm, blastoderm-yolk (BYI) and yolk interfaces have surface tensions T_{b}, $T_{\mathrm{byi}}$and T_{y}, and the ratio of yolk to blastoderm volume is conserved.

(C) Force balance and contact angles at the contact line where the three interfaces shown in (A) meet.

(D) Schematic of compression experiment. A force with magnitude $|\boldsymbol{F}|$ is exerted on the embryo through compression with the upper plate. The mass density of the embryo ρ results in an force on the lower plate with increased magnitude, $|\boldsymbol{F_l}| > |\boldsymbol{F}|$.

(E) Dynamic model of zebrafish doming, where the blastoderm and EVL are represented by active viscous fluids. The angular coordinate around the animal-vegetal axis is denoted ϕ, and the embryo is assumed to be axisymmetric around the animal-vegetal axis. A coordinate going along the embryo of the surface from the animal pole is denoted s. $\bar{\boldsymbol{e}}_s$, $\bar{\boldsymbol{e}}_\phi$and $\bar{\boldsymbol{n}}$ are the tangent and normal vectors to the blastoderm surface, respectively. The blastoderm flow field is denoted $\boldsymbol{v}$ and the EVL flow field $\bar{\boldsymbol{v}}$. The stress tensor inside the blastoderm is denoted $\sigma_{\alpha\beta}$. The active anisotropic part of the stress tensor $\zeta_{\alpha\beta}$ is assumed to be radially oriented away from the center of the embryo.

(F and G) Simulated contact angles at the contact line corresponding to simulations shown in Figures 2E and 2F. Simulations were stopped when the angle between the EVL and the YSL approached a zero value. Continuous lines correspond to experimental measurements in WT embryos. In the extreme scenarios considered here, simulated and experimental contact angles do not agree (compare with Figures 4D-4F). Error bars, ± s.d.

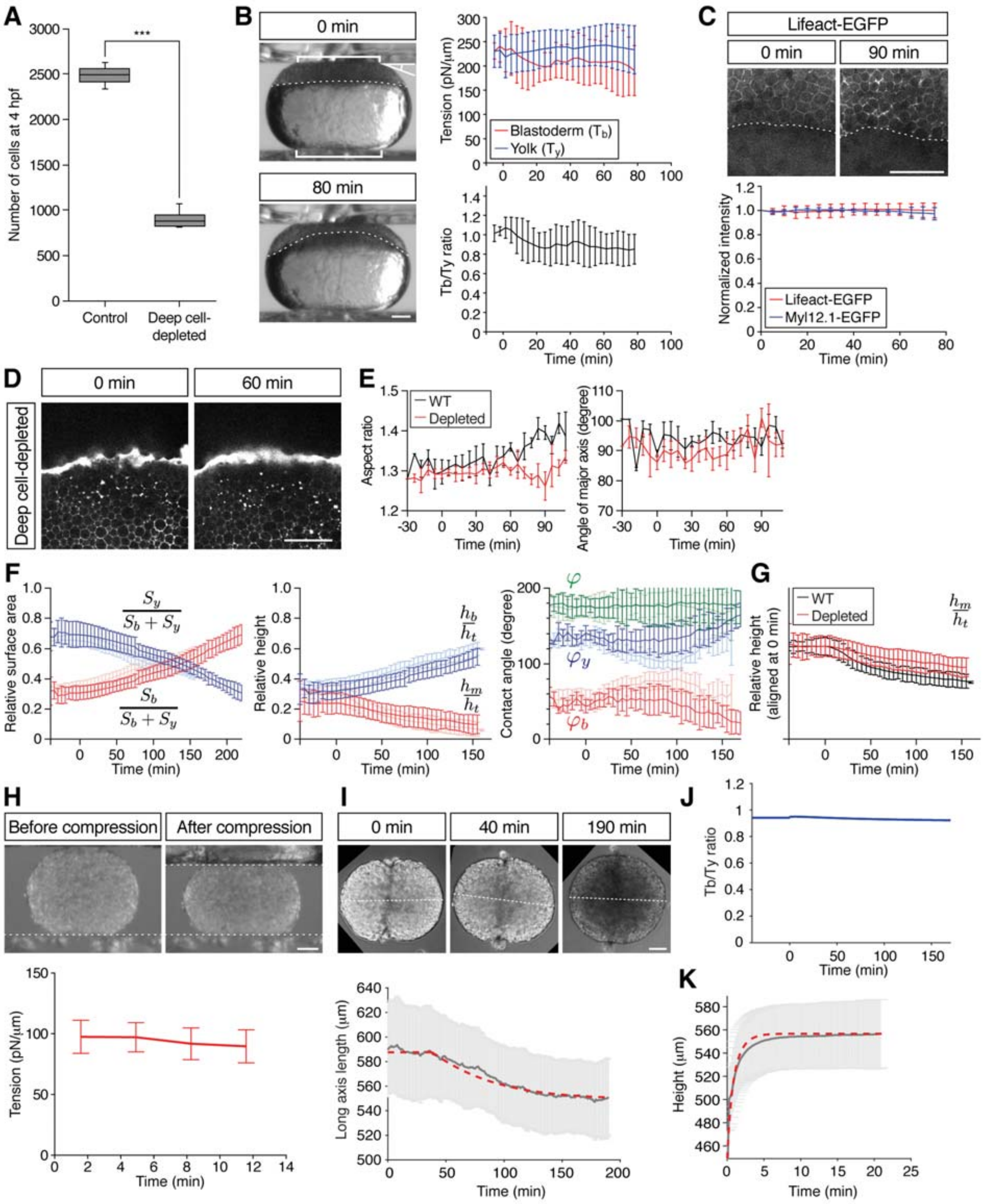

Figure S3. Characterization of Deep Cell-Depleted Embryos and Tissue Explants, Related to Figure 4

(A) Number of deep cells in WT control and deep cell-depleted embryos. Confocal images of sphere stage (4 hpf) embryos with nuclei marked by H2A-mCherry expression at the center of the embryo (400×400×300 μm volume) were used to count deep cells. Box plots represent 5%, 25%, median, 75% and 95%. ****P* < 0.001 (*t*-test). *n* embryos = 3 (control) and 5 (deep cell-depleted).

(B) Measurement of blastoderm (T_b) and yolk cell surface tension (T_y) in deep cell-depleted WT embryos during the course of doming using tissue tensiometry. Embryos were compressed by 20% of their initial

(legend continued on next page)

uncompressed height. Panels on the left are bright-field images of a deep cell-depleted embryo before (0 min; top) and after doming (+80 min; bottom). Brackets, contact areas. Kinked line, contact angle. Dashed lines, BYI. Panels on the right show quantification of T_b (top, red line) and T_y (top, blue line) and T_b/T_y (bottom, black line) as a function of time after compression. *n* embryos = 7. Error bars, ± s.d. Scale bar, 100 μm.

(C) Actin and myosin II localization at the BYI. Two-photon microscopy sections of the BYI region at ~100 μm depth from the surface of a *Tg(actb1:Lifeact-EGFP)* embryo at the onset (upper left) and end (upper right) of doming. Animal pole is up. EGFP signal intensity within a ~100 μm region of the YSL below the BYI of *Tg(actb1:Lifeact-EGFP)* (red line) and *Tg(actb1:Myl12.1-EGFP)* (blue line) embryos were quantified during doming and normalized by the signal intensity at 0 min (bottom). *n* embryos = 12 (Lifeact-EGFP) and 6 (Myl12.1-EGFP). Dotted lines, BYI. Error bars, ± s.d. Scale bar, 100 μm.

(D) Yolk granule shape in deep cell-depleted WT embryos. Single plane confocal images of yolk granules in a deep cell-depleted embryo injected with fluorescent dextran into the yolk at 0 min (left) and +60 min (right) of doming. Scale bar, 100 μm.

(E) Quantification of yolk granule aspect ratio (left) and angle of major axis (right) from -30 to +108 min of doming in intact WT (black) and deep cell-depleted embryos. *n* embryos = 4 (intact) and 6 (deep cell-depleted). Error bars, ± s.e.m.

(F) Comparison of WT control with deep cell-depleted embryo shapes (surface area, height and contact angles) during doming. Pale red and blue lines show WT control embryos, and dark red and blue lines deep cell-depleted embryos. *n* embryos = 6 (control) and 5 (deep cell-depleted). Error bars, ± s.d.

(G) Comparison of relative blastoderm height in WT (black line) and deep cell-depleted (red line) embryos during doming. The relative height data were taken from the middle panel of Figure S3F (h_m/h_t) and aligned at the onset of doming (0 min) to better illustrate subsequent changes between WT and deep cell-depleted embryos during the course of doming. Error bars, ± s.d.

(H) Measurement of surface tension of blastoderm explants. Fused blastoderm explants were compressed by 20% of their initial uncompressed height. Upper panels show the explant before (upper left) and after (upper right) compression. Dotted lines, surface position of the upper and lower plates. Lower panel is the quantification of the surface tension of the explants. t = 0 min corresponds to the onset of doming in unperturbed control embryos. *n* = 4. Error bars, ± s.d. Scale bar, 100 μm.

(I) Fusion of blastoderm explants. Two blastoderm explants consisting of EVL and deep cells were removed from 1k-cell stage embryos (3 hpf) and put into contact with each other. Changes in the length of the longest axis of the fused explants were monitored as a function of time during fusion. Upper panels are still images from a time-lapse movie of fusing explants. Dotted lines, measured long axis length. Lower panel shows the quantification of the long axis length, in which pale grey lines show experimental results, while dashed thick red lines show the outcome of simulations of this process using a sphere relaxing to its equilibrium shape after equatorial deformation as a proxy for the fusion process (see also Methods S1). The first ~ 40 min have not been taken into account in the fitting procedure in order to avoid artifacts due to wound healing at early stages of explant fusion. The values of the viscosities of deep cell layer and EVL used for the simulation are specified in Table S1C. t = 0 min corresponds to about 20-30 min later after the onset of fusion. *n* explants = 9. Error bars, ± s.d. Scale bar, 100 μm.

(J) Calculated ratio of blastoderm to yolk total surface tension in simulations of deep-cell depleted embryos.

(legend continued on next page)

(K) Experimental measurements and simulations of yolk cell relaxation after compression to determine yolk cell viscosity. Yolk explants were obtained from embryos at the 256- and 512-cell stages (2.5 - 2.75 hpf) and compressed when unperturbed control embryos had reached the onset of doming. After 30 min of compression, the upper plate was removed and the relaxation of the yolk explant height was measured. Pale gray line represents the experimental results and red dashed line shows the outcome of the simulations of this process. The value of yolk cell viscosity used to fit the simulations to the experimental results = 40 Pa s. *n* explants = 17. Error bars, ± s.d.

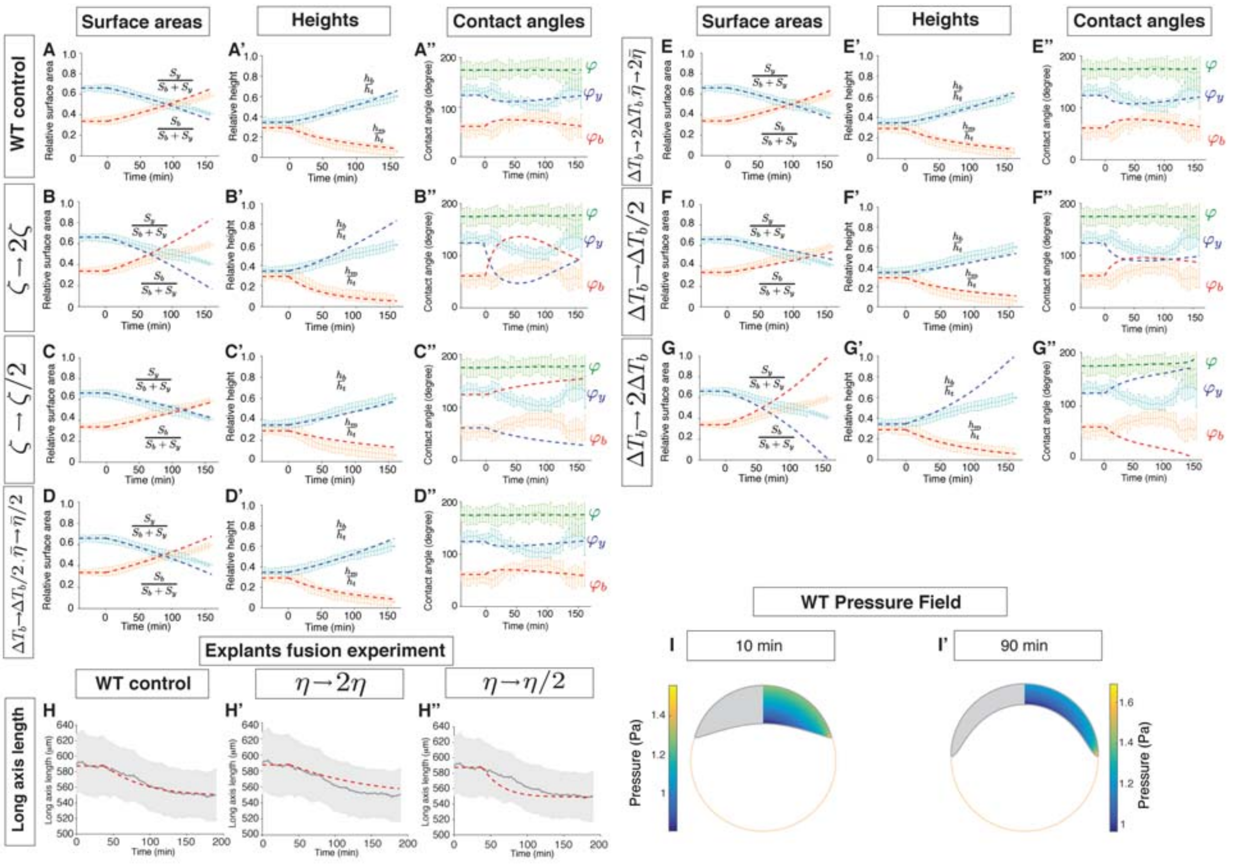

Figure S4. Sensitivity of the Dynamical Doming Model to Changes in Tissue Viscosity, Active Stress, and EVL Surface Tension Reduction, Related to Figure 4

(A-G'') Simulations of embryo surface areas (A-G), heights (A'-G') and contact angles (A''-G'') as a function of time during doming with pale blue and red curves showing the experimental measurements, and dashed red and blue thick lines showing simulation results. In (A-A'') the same parameters as in Figures 4F-4F'' were used for simulations. In (B-B'') the blastoderm active stress was increased by a factor of 2. In (C-C'') the blastoderm active stress was reduced by a factor of 2. In (D-D''), the EVL viscosity and blastoderm surface tension reduction were both reduced by a factor of 2. In (E-E''), the EVL viscosity and blastoderm surface tension reduction were both increased by a factor of 2. In (F-F'') the blastoderm surface tension reduction was reduced by a factor 2. In (G-G'') the blastoderm surface tension reduction was increased by a factor 2.

(H-H'') Simulations of explant relaxation after fusion (see also Figure S3I). Pale grey lines show experimental results while red thick dashed lines the outcome of simulations. In (H) the same parameters as in Figure S3I were used. In (H') and (H'') the blastoderm viscosity was increased or decreased by a factor 2 with respect to its value in (H), respectively.

(I and I') Simulation of the pressure field in a WT embryo at 10 min (I) and 90 min (I') after the onset of doming. Scale bars show color coding of pressure in Pa.

Error bars, ± s.d.

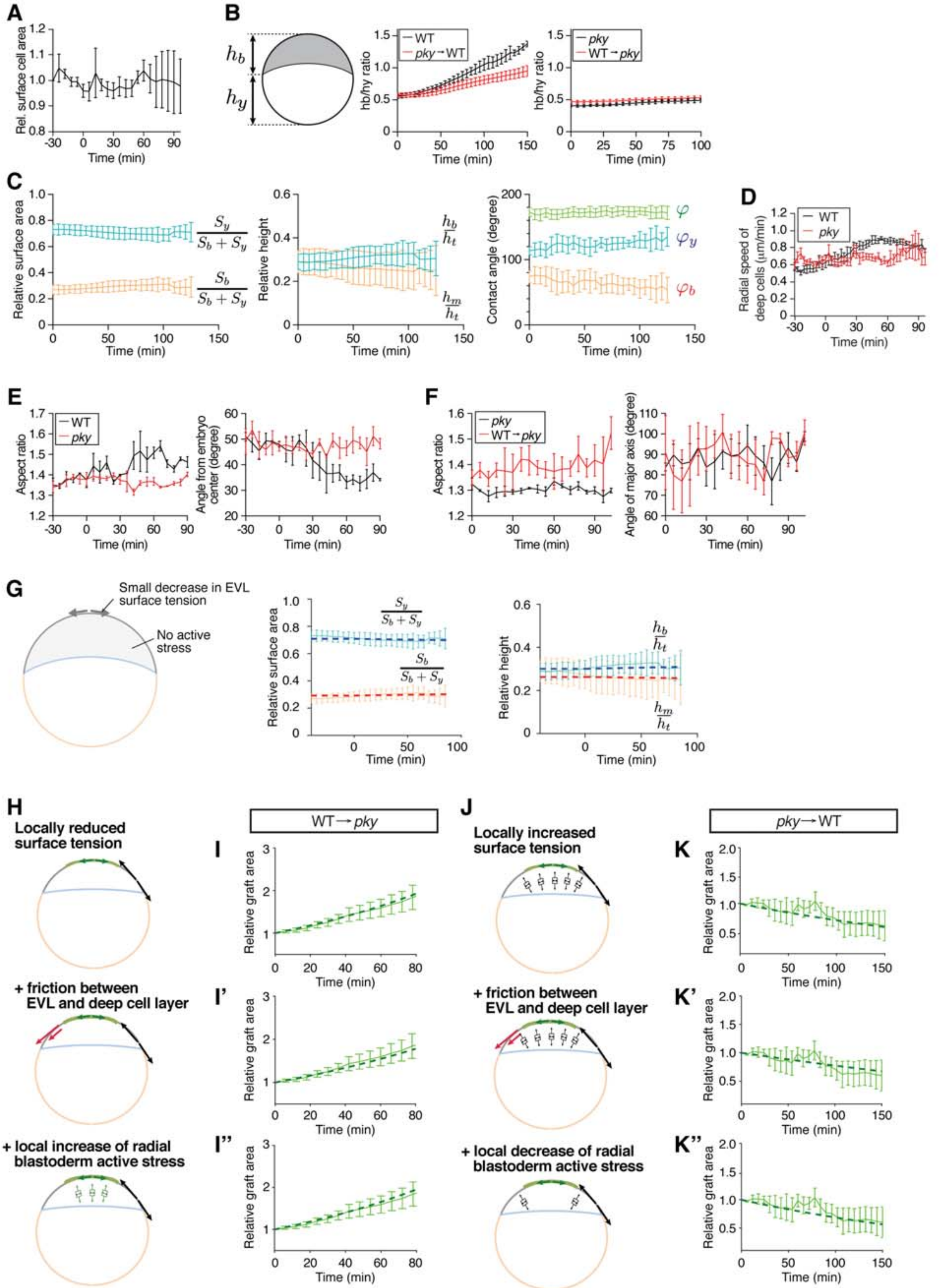

Figure S5. Tissue and Cell Shape Changes and Simulations of the Doming Phenotype in *pky* Mutants and EVL/Surface Cell-Transplanted Embryos, Related to Figures 5 and 6

(A) Surface area of deep cell-depleted *pky* embryos. *n* embryos = 3. Error bars, ± s.e.m.

(legend continued on next page)

(B) Ratio of the distance between the animal pole and contact line (h_b) to the distance between contact line and the vegetal pole (h_y) as a measurement for the position of the contact line. *n* embryos = 6 (WT), 3 (transplantation *pky* to WT), 4 (*pky*) and 3 (transplantation WT to *pky*). Error bars, ± s.d.

(C) Geometrical parameters of *pky* embryos during doming with relative surface area, relative height and contact angles quantified from bright-field embryo images. *n* embryos = 4. Error bars, ± s.d.

(D) Comparison of deep cell radial movements between WT (black line) and *pky* mutant (red line) embryos. *n* embryos = 4. Error bars, ± s.e.m.

(E) Comparison of deep cell shape (left panel, aspect ratio; right panel, angle from embryo center) between WT and *pky* mutant embryos. *n* embryos = 3. Error bars, ± s.e.m.

(F) Aspect ratio (left) and angle of major axis (right) of yolk granules in intact *pky* embryos (black line) and *pky* embryos with transplanted WT EVL (red line). t = 0 min corresponds to sphere stage (4 hpf). *n* embryos = 3 for *pky* and EVL-transplanted *pky* each. Error bars, ± s.e.m.

(G) Simulations for the doming defect in *pky* mutants. A combination of strongly impaired EVL surface tension reduction and absent deep cell layer contraction gives rise to embryo shapes resembling *pky* mutant embryos during doming. Right panels are plots of embryo surface area and height as a function of time during doming with pale blue and red curves showing the experimental measurements, and dashed red and blue thick lines showing simulation results. Simulations parameters are specified in Table S1C. Error bars, ± s.d.

(H and J) Schematic of possible mechanisms involved in rescuing doming by transplanting WT EVL cells on *pky* embryos (H), or inhibiting doming by transplanting *pky* surface cells on WT embryos (J).

(I-I'', K-K'') Simulation of surface area of the grafted patch in either *pky* embryo shapes as a result of WT EVL transplantations (I-I") or WT embryo shapes as a result of *pky* surface cell transplantations (K-K"), according to the three mechanisms described in (H) and (J). Pale green curves, experimental measurements, dashed green thick lines, simulation results. Error bars, ± s.d.

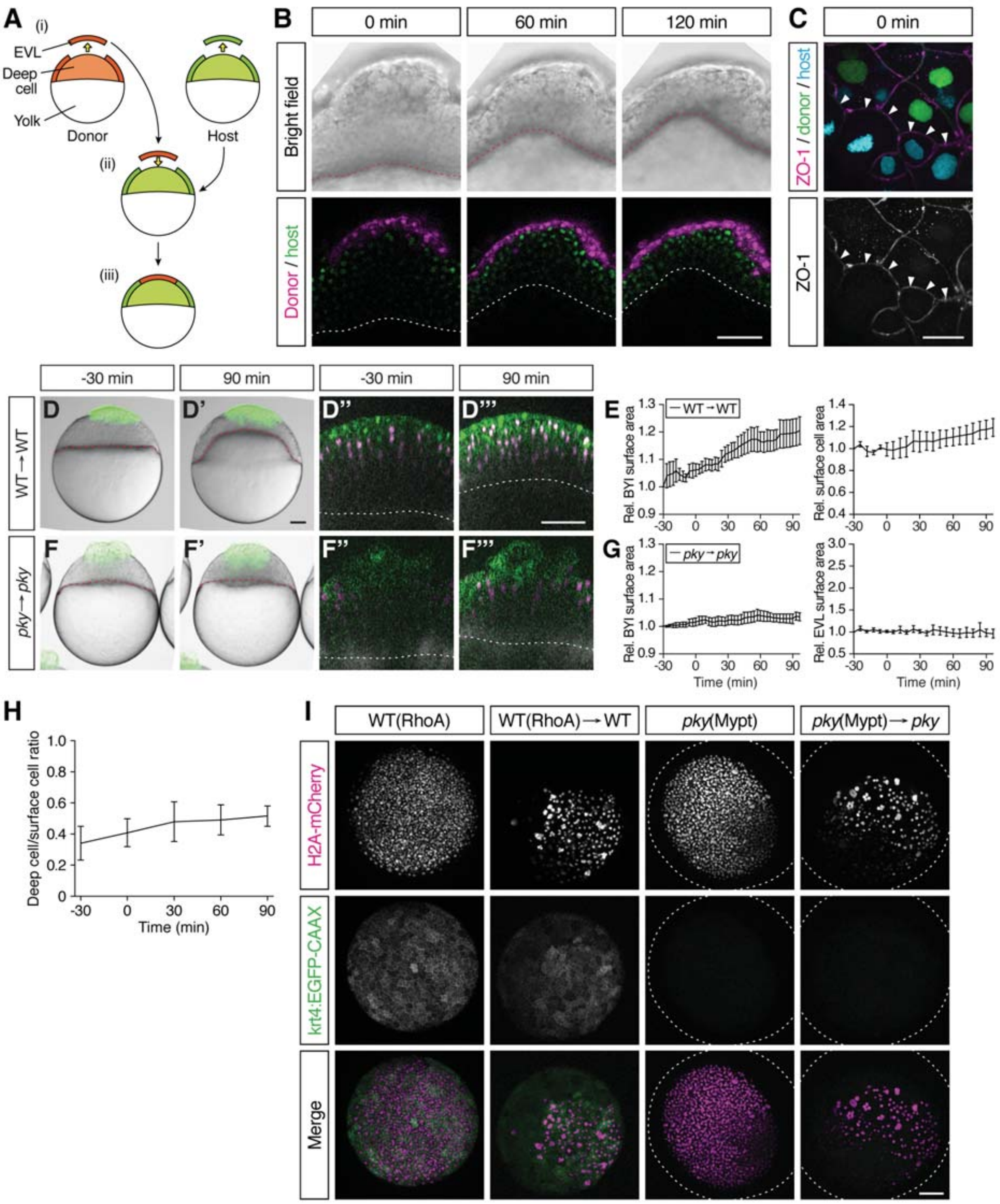

Figure S6. Characterization of Embryo Shapes and Surface Cell Differentiation in the Transplantation Experiments, Related to Figure 5

(A) Schematic of the procedure of EVL/surface cell transplantations. Donor and host embryos are labeled with fluorescent proteins or dyes of different color. ≈ 70 EVL/surface cells are taken from the donor embryo and nearly the same number of EVL/surface cells was removed from the host embryo (i); donor EVL/surface cells are placed on the surface-cell free area of the host embryo (ii and iii).

(B) Bright-field (upper row) and confocal microscopy images (bottom row) of a WT embryo containing transplanted cells from a WT donor embryo at the onset (0 min), middle (60 min) and end of doming (120

(legend continued on next page)

min). There are only very few deep cells from the donor embryo compared with the number of transplanted EVL cells. The plasma membrane and nuclei of the transplanted EVL cells are marked by mem-RFP (magenta) and H2A-mCherry (magenta), respectively. The plasma membrane and nuclei of the host cells were marked with mem-GFP (green) and H2B-GFP (green), respectively. Red and white dotted lines, BYI. Scale bar, 100 μm.

(C) Tight junction formation, marked by the localization of the tight junction component ZO-1, between transplanted WT donor cells and *pky* mutant host cells (*n* embryos = 11). ZO-1 (white) is localized to the interface between donor (green nuclei; WT) and host cells (cyan nuclei; *pky*) indicative of tight junction formation at the interface between these cells. Scale bar, 20 μm.

(D-G) Embryos where EVL/surface cells were transplanted from WT to WT embryos (D-E) (*n* embryos = 3) and from *pky* to *pky* embryos (F-G) (*n* embryos = 3). Bright-field images of mosaic embryos before (-30 min) and after completion of doming (+90 min) with transplanted cells marked by fluorescent dextran (green; D, D', F and F'). Confocal images with plasma membrane expressing mem-GFP (green), nuclei marked by H2A-mCherry (magenta) and BYI outlined by fluorescent dextran (white; D'', D''', F'' and F'''). Transplanted cells were marked by fluorescent dextran (green; D, D', F and F') or H2B-GFP (green; D'', D''', F'' and F'''). Red and white dashed lines, BYI. Changes in relative BYI area (left column) and relative EVL/surface cell area (right column) as a function of time during doming in the different transplantation experiments (E and G). Error bars, ± s.e.m. Scale bars, 100 μm.

(H) Ratio of deep-to-surface cells for transplanted RhoA-overexpressing cells as a function of time during doming (*n* embryos = 4). t = 0 min corresponds to the onset of doming. Error bars, ± s.e.m.

(I) EVL differentiation in RhoA- or Mypt1-overexpressing embryos. RhoA-overexpressing WT EVL cells in intact (top row) and transplanted (second row from top) embryos expressing the EVL-differentiation marker *krt4*:EGFP-CAAX. Mypt1-overexpressing *pky* cells in intact (third row from top) and transplanted embryos (bottom row) do not show *krt4*:EGFP-CAAX expression. *H2A-mCherry* mRNA was co-injected with *RhoA* or *mypt1* mRNA into one-cell stage embryo to mark the injected cells. Dotted lines, embryo outline. Scale bar, 100 μm.

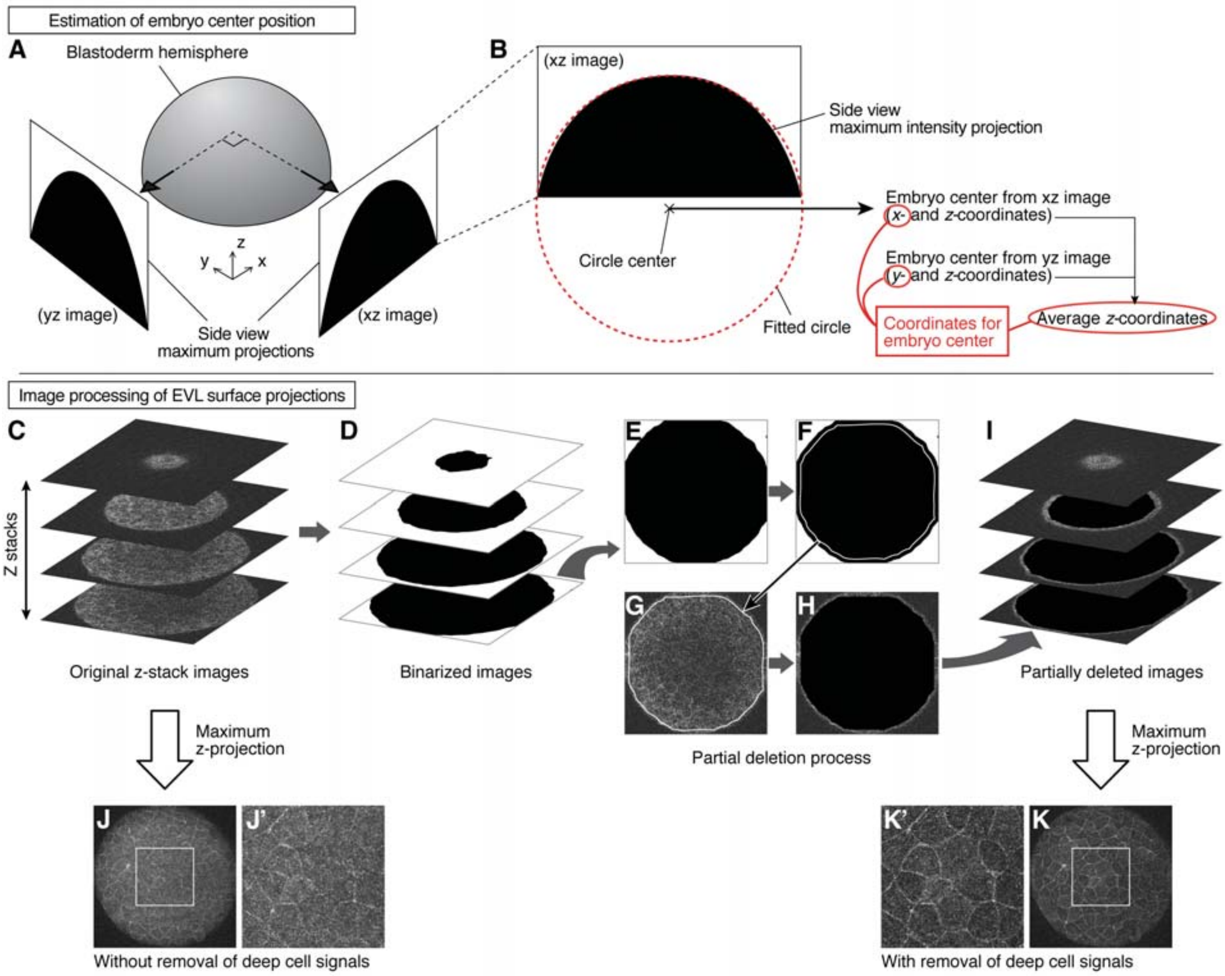

Figure S7. Estimation of Embryo Center Position and Measurement of EVL Surface Projections, Related to STAR Methods

(A and B) Estimation of embryo center position. Side views of the animal pole hemisphere were obtained from two perpendicular directions in the two-photon microscope stack as maximum intensity projections (A). A circle was fitted along the arc of this hemisphere projection (red dotted line), and the center of the fitted circle was used to estimate the embryo center (B).

(C-K) Image processing of EVL surface projections. Z-stack images obtained by two-photon microscopy were first processed by binarization (C and D). The outline of each image slice was detected and its size was reduced by the expected thickness of surface cells (E and F). The detected and reduced outline was applied to the original image slice, and the inner area of this outline was filled with black color (G and H). After processing the entire stack with this method, they were superimposed by maximum intensity projection (I). The resultant projection image shows clearer surface cell outlines compared to a projection image from an unprocessed stack. (J-K’). White rectangle regions in (J) and (K) are magnified in (J’) and (K’), respectively.

Supplemental Table Legend

Table S1. Values from Experimental Measurements and Lists of Parameters Used in The Simulations, Related to Figures 2, 4, 6, S2, S3, S4 and S5

(A) Measurements of geometrical properties of embryos, averaged over 10 minutes prior to the onset of doming. Errors are standard deviations. WT, wild-type control embryos (n = 6); Depleted, deep cell-depleted embryos (n = 5, see main text for details).

(B) Experimental measurement of embryo sinking velocity in Danieau's medium at 23 ºC. The density of Danieau's medium was determined by weighting 1L of medium, and its viscosity was assumed to be equal to the viscosity of water at the same temperature. WT, wild-type control embryos; Depleted, deep cell-depleted embryos (see main text for details).

(C) Parameter list for finite element simulations of doming. t = 0 corresponds to the onset of doming. In these simulations the friction coefficient ξ is taken to be equal to 0. Simulations shown in Figures 2E and 2F are performed for illustrative purposes. WT, wild-type control embryos; Depleted, deep cell-depleted embryos. The yolk surface tension for *pky* embryos was determined from compression experiments as described in section 2 (data not shown), and the ratio of surface tensions prior to doming were assumed to be identical in *pky* mutant and WT embryos.

(D) Parameter list for finite element simulations of the grafting experiments.

Supplemental Movie Legends

Movie S1. Embryo Doming, Related to Figure 1

Bright-field time-lapse movie of a WT zebrafish embryo from pre-doming stage (-30 min) to the end of doming (+90 min). 120 min total with 10 min time intervals. Animal, top and vegetal, bottom. Scale bar, 100 μm.

Movie S2. Simulations of Embryo Doming, Related to Figure 2

Simulations of embryo doming for the two different scenarios hypothesized in Figure 2 where either reduced blastoderm surface tension (i; Figure 2E) or increased deep cell layer contraction (ii; Figure 2F) drive doming. 160 (i) and about 80 (ii) min total with 2 min and 1 min time intervals, respectively.

Movie S3. Deep Cell Movement and Polarization during Doming, Related to Figure 3

Confocal time-lapse movie of deep cells directly below the EVL near the animal pole region of a *Tg(actb1:lifeact-GFP)* embryo during doming. 90 min total with 1 min time intervals. Asterisks, EVL cells. Animal (EVL cells), top and vegetal, bottom. Scale bar, 10 μm.

Movie S4. Doming of Deep Cell-Depleted Embryo, Related to Figure 4

(i) Bright-field time-lapse movie of a deep cell-depleted embryo from pre-doming stage (-30 min) to the end of doming (+90 min). 120 min total with 10 min time intervals. Animal, top and vegetal, bottom. Scale bar, 100 μm.

(ii) Single-plane confocal time-lapse movie of a deep cell-depleted WT embryo from pre-doming stage (-39 min) until after completion of doming (+210 min). Plasma membrane and nuclei are labeled by mem-GFP (green) and H2A-mCherry (magenta), respectively, and BYI is marked by fluorescent dextran (white). 249 min total with 6 min time intervals. Animal, top and vegetal, bottom. Scale bar, 100 μm.

(iii and iv) Simulations movies of embryo doming respectively for deep-cell depleted embryos (iii) and WT control embryos (iv). 160 min total with 2 min time intervals

Movie S5. Doming in EVL/surface Cell Transplanted Embryos, Related to Figures 5 and 7

Time-lapse movies with overlay of bright-field (gray, donor embryo) and fluorescent (green, transplanted cells) images of mosaic embryos where *pky* surface cells were transplanted in WT (i), WT EVL cells overexpressing RhoA in WT (ii) and WT EVL cells in *pky* embryos (iii). 120 min total with 10 min time intervals for each sample. Animal, top and vegetal, bottom. Scale bar, 100 μm.

Movie S6. Simulations of Doming in EVL/surface Cell Transplanted Embryos, Related to Figure 6

Simulations of doming in WT embryos on which a patch of expansion-defective *pky* mutant surface cells was transplanted (i; Figure 6B”), and of *pky* mutant embryos on which a patch of expansion-competent WT EVL cells was transplanted (ii; Figure 6D”). 80 min (i) and 180 min (ii) total with 2 min time intervals.

Methods S1. Supplementary Theory, Related to STAR Methods

SUPPLEMENTARY THEORY

We propose a theoretical description of the shape, shape changes and cellular flows occurring during doming in the zebrafish embryo. In this description, deep cells are modelled as a 3D incompressible active viscous fluid, the EVL epithelium as a thin layer of active, 2D compressible viscous fluid, under surface tension. Such an active fluid description captures the viscous resistance to flow of the tissue as well as autonomous cellular force generation within the tissue [Prost et al., 2015, Ranft et al., 2010, Etournay et al., 2015, Behrndt et al., 2012]. The yolk cell is described as a membrane with a surface tension, filled with a fluid of negligible viscosity compared to other dissipation processes occurring during doming.

In the first part of the these supplements, we calculate static equilibrium shapes of the embryo predicted by this description. We then describe compression experiments of zebrafish embryos and how surface tensions can be extracted from force measurements of compressed embryos. In the last part, we propose a dynamic description of doming, taking into account the viscosities of the blastoderm and EVL, and internal autonomous active stresses in the blastoderm and EVL. We then describe finite element simulations of the dynamical equations.

1. Surface tension description of the zebrafish embryo

We start by discussing the equilibrium shape of a model of the Zebrafish embryo based on surface tensions. We consider here that the blastoderm and yolk have a fixed volume. The blastoderm and yolk have interfaces with the external medium, with respective surface tension T_{b} and T_{y} (Figure S2B). The surface tension of the blastoderm corresponds to the surface tension of the enveloping layer (EVL), a thin epithelium covering the blastoderm. In addition, the blastoderm and yolk have a common interface, denoted blastoderm-yolk interface (BYI) with interfacial tension T_{byi}. The pressures within the blastoderm and the yolk are uniform and are denoted P_{y} and P_{b}. The law of Laplace then implies that these interfaces are portions of sphere, and the overall shape has rotational symmetry around an axis of symmetry joining the poles of the blastoderm (animal pole) and of the yolk (vegetal pole).

1.1. **Force balance equations.** The mechanical work of the surface tension-based model can be written

$$W = T_{\mathrm{y}} S_{\mathrm{y}} + T_{\mathrm{b}} S_{\mathrm{b}} + T_{\mathrm{byi}} S_{\mathrm{byi}} - P_{\mathrm{y}}(V_{\mathrm{y}} - V_{\mathrm{y}}^0) - P_{\mathrm{b}}(V_{\mathrm{b}} - V_{\mathrm{b}}^0) \quad (1)$$

with S_{y} the surface area of the yolk/medium interface, S_{b} the surface area of the blastoderm/medium interface, S_{byi} the surface area of the BYI, V_{y} the volume of the yolk, V_{b} the volume of the blastoderm, V_{y}^0 the preferred volume of the yolk and V_{b}^0 the preferred volume of the blastoderm.

To find the equilibrium shape, we note that the shape of the embryo can be described in terms of 4 lengths: the heights h_{b}, h_{c} and h_{y} and the radius of the contact circle r_{c} where the yolk, blastoderm and BYI meet (Figure S2B). The surface areas and volumes relate to these lengths according to

$$S_{\mathrm{y}} = \pi(r_{\mathrm{c}}^2 + h_{\mathrm{y}}^2) \tag{2}$$

$$S_{\mathrm{b}} = \pi(r_{\mathrm{c}}^2 + h_{\mathrm{b}}^2) \tag{3}$$

$$S_{\mathrm{c}} = \pi(r_{\mathrm{c}}^2 + h_{\mathrm{c}}^2) \tag{4}$$

$$V_{\mathrm{y}} = \frac{\pi}{6}\left[h_{\mathrm{y}}(3r_{\mathrm{c}}^2 + h_{\mathrm{y}}^2) + h_{\mathrm{c}}(3r_{\mathrm{c}}^2 + h_{\mathrm{c}}^2)\right] \tag{5}$$

$$V_{\mathrm{b}} = \frac{\pi}{6}\left[h_{\mathrm{b}}(3r_{\mathrm{c}}^2 + h_{\mathrm{b}}^2) - h_{\mathrm{c}}(3r_{\mathrm{c}}^2 + h_{\mathrm{c}}^2)\right] \tag{6}$$

Plugging these expressions in the mechanical work Eq. 1 and differentiating with respect to r_{c}, h_{y}, h_{b} and h_{c}, one obtains the force balance equations

$$2(T_{\mathrm{y}} + T_{\mathrm{b}} + T_{\mathrm{byi}}) = P_{\mathrm{y}}(h_{\mathrm{y}} + h_{\mathrm{c}}) + P_{\mathrm{b}}(h_{\mathrm{b}} - h_{\mathrm{c}}) \tag{7}$$

$$P_{\mathrm{y}} = 2T_{\mathrm{y}}\frac{2h_{\mathrm{y}}}{r_{\mathrm{c}}^2 + h_{\mathrm{y}}^2} \tag{8}$$

$$P_{\mathrm{b}} = 2T_{\mathrm{b}}\frac{2h_{\mathrm{b}}}{r_{\mathrm{c}}^2 + h_{\mathrm{b}}^2} \tag{9}$$

$$P_{\mathrm{y}} - P_{\mathrm{b}} = 2T_{\mathrm{byi}}\frac{2h_{\mathrm{c}}}{r_{\mathrm{c}}^2 + h_{\mathrm{c}}^2} \tag{10}$$

where the three last equations correspond to the law of Laplace expressed at different interfaces, as can be seen from the expression of the radii of curvature $R_{\mathrm{y}} = (r_{\mathrm{c}}^2 + h_{\mathrm{y}}^2)/(2h_{\mathrm{y}})$, $R_{\mathrm{b}} = (r_{\mathrm{c}}^2 + h_{\mathrm{b}}^2)/(2h_{\mathrm{b}})$, and $R_{\mathrm{c}} = (r_{\mathrm{c}}^2 + h_{\mathrm{c}}^2)/(2h_{\mathrm{c}})$. Eliminating the pressure in equations 7 and 10 yields the two force balance equations:

$$T_{\mathrm{y}}\frac{r_{\mathrm{c}}^2 - h_{\mathrm{y}}^2}{r_{\mathrm{c}}^2 + h_{\mathrm{y}}^2} + T_{\mathrm{b}}\frac{r_{\mathrm{c}}^2 - h_{\mathrm{b}}^2}{r_{\mathrm{c}}^2 + h_{\mathrm{b}}^2} + T_{\mathrm{byi}}\frac{r_{\mathrm{c}}^2 - h_{\mathrm{c}}^2}{r_{\mathrm{c}}^2 + h_{\mathrm{c}}^2} = 0, \tag{11}$$

$$T_{\mathrm{b}}\frac{h_{\mathrm{b}}}{r_{\mathrm{c}}^2 + h_{\mathrm{b}}^2} - T_{\mathrm{y}}\frac{h_{\mathrm{y}}}{r_{\mathrm{c}}^2 + h_{\mathrm{y}}^2} + T_{\mathrm{byi}}\frac{h_{\mathrm{c}}}{r_{\mathrm{c}}^2 + h_{\mathrm{c}}^2} = 0. \tag{12}$$

Fixing in addition the volume of the yolk and the blastoderm in Eqs. 5 and 6 then yields a system of 4 equations which can be solved for the 4 unknowns h_{b}, h_{c}, h_{y} and r_{c}.

We note that the three contact angles at the contact line separating the EVL, yolk surface and BYI are related to the geometrical parameters h_{b}, h_{y}, h_{c} and r_{c} by $\varphi_b = \theta_b - \theta_c$, $\varphi_{\mathrm{y}} = \theta_{\mathrm{y}} + \theta_{\mathrm{c}}$, $\varphi = 2\pi - \theta_{\mathrm{b}} - \theta_{\mathrm{y}}$ with

$$\cos\theta_{\mathrm{b}} = \frac{r_{\mathrm{c}}^2 - h_{\mathrm{b}}^2}{r_{\mathrm{c}}^2 + h_{\mathrm{b}}^2} \quad , \sin\theta_{\mathrm{b}} = \frac{2h_{\mathrm{b}}r_{\mathrm{c}}}{r_{\mathrm{c}}^2 + h_{\mathrm{b}}^2} \tag{13}$$

$$\cos\theta_{\mathrm{y}} = \frac{r_{\mathrm{c}}^2 - h_{\mathrm{y}}^2}{r_{\mathrm{c}}^2 + h_{\mathrm{y}}^2} \quad , \sin\theta_{\mathrm{y}} = \frac{2h_{\mathrm{y}}r_{\mathrm{c}}}{r_{\mathrm{c}}^2 + h_{\mathrm{y}}^2} \tag{14}$$

$$\cos\theta_{\mathrm{c}} = \frac{r_{\mathrm{c}}^2 - h_{\mathrm{c}}^2}{r_{\mathrm{c}}^2 + h_{\mathrm{c}}^2} \quad , \sin\theta_{\mathrm{c}} = \frac{2h_{\mathrm{c}}r_{\mathrm{c}}}{r_{\mathrm{c}}^2 + h_{\mathrm{c}}^2} \tag{15}$$

One can then verify that the balance equations 11 and 12 are equivalent to

$$T_{\mathrm{y}} + T_{\mathrm{byi}}\cos\varphi_{\mathrm{y}} + T_{\mathrm{b}}\cos\varphi = 0, \tag{16}$$

$$T_{\mathrm{byi}}\sin\varphi_{\mathrm{y}} - T_{\mathrm{b}}\sin\varphi = 0, \tag{17}$$

which correspond to the balance of surface tensions at the contact line (Figure S2C).

1.2. **Solutions for embryo equilibrium shapes.** To solve Eqs. 11-12, we introduce a characteristic length R_t, verifying $\frac{4}{3}\pi R_t^3 = V_{\mathrm{b}} + V_{\mathrm{y}}$ the total volume of the embryo. We then define the adimensional lengths $\bar{h}_{\mathrm{y}} = h_{\mathrm{y}}/R_t$, $\bar{h}_{\mathrm{b}} = h_{\mathrm{b}}/R_t$, $\bar{h}_{\mathrm{c}} = h_{\mathrm{c}}/R_t$, $\bar{r}_{\mathrm{c}} = r_{\mathrm{c}}/R_t$, and the volume fractions of the blastoderm and of the yolk $v_{\mathrm{b}} = V_{\mathrm{b}}/(V_{\mathrm{b}}+V_{\mathrm{y}})$ and $v_{\mathrm{y}} = 1 - v_{\mathrm{b}}$. Solving the volume equations Eqs. 5 and 6 for the heights $\bar{h}_{\mathrm{y}}$ and $\bar{h}_{\mathrm{b}}$ then yields the solution

$$\bar{h}_{\mathrm{y}} = \bar{r}_{\mathrm{c}} \times g\left(\frac{8v_{\mathrm{y}} - \bar{h}_{\mathrm{c}}(3\bar{r}_{\mathrm{c}}^2 + \bar{h}_{\mathrm{c}}^2)}{\bar{r}_{\mathrm{c}}^3}\right) \tag{18}$$

$$\bar{h}_{\mathrm{b}} = \bar{r}_{\mathrm{c}} \times g\left(\frac{8v_{\mathrm{b}} + \bar{h}_{\mathrm{c}}(3\bar{r}_{\mathrm{c}}^2 + \bar{h}_{\mathrm{c}}^2)}{\bar{r}_{\mathrm{c}}^3}\right) \tag{19}$$

with

$$g(x) = (-2 + 2^{1/3}\left(x + \sqrt{4 + x^2}\right)^{2/3})/(2^{2/3}\left(x + \sqrt{4 + x^2}\right)^{1/3}) \tag{20}$$

a mathematical function. Imposing the value of $v_{\mathrm{b}} = 1 - v_{\mathrm{y}}$, as well as the tension ratios $T_{\mathrm{b}}/T_{\mathrm{y}}$ and $T_{\mathrm{byi}}/T_{\mathrm{y}}$, and plugging Eqs. 18 and 19 in the force balance equations 11 and 12, we then solve numerically for the values of r_{c} and h_{c}. The corresponding solutions are plotted for $v_{\mathrm{b}} = 0.235$ in Figure 2B in the main text, as a function of the tension ratios $T_{\mathrm{b}}/T_{\mathrm{y}}$ and $T_{\mathrm{byi}}/T_{\mathrm{y}}$.

Above critical values of the tension ratios $T_{\mathrm{b}}/T_{\mathrm{y}}$ and $T_{\mathrm{byi}}/T_{\mathrm{y}}$, no solution can be found. These situation correspond to three extreme cases:

- (i) $T_{\mathrm{y}} > T_{\mathrm{b}} + T_{\mathrm{byi}}$: engulfment of the yolk in the blastoderm.
- (ii) $T_{\mathrm{b}} > T_{\mathrm{y}} + T_{\mathrm{byi}}$: engulfment of the blastoderm in the yolk.

- (iii) $T_{\rm byi} > T_{\rm b} + T_{\rm y}$: separation of the yolk and blastoderm.

Condition (i) can be found by noting that Eqs. 11 and 12 can be combined to yield

$$T_{\rm y} = T_{\rm b}\frac{h_{\rm b}^2 - r_{\rm c}^2 + 2h_{\rm b}h_{\rm y}}{h_{\rm b}^2 + r_{\rm c}^2} + T_{\rm byi}\frac{h_{\rm c}^2 - r_{\rm c}^2 + 2h_{\rm y}h_{\rm c}}{h_{\rm c}^2 + r_{\rm c}^2}. \tag{21}$$

Letting $r_{\rm c} \to 0$ and $h_{\rm y} \to 0$ in the equation above, corresponding to vanishing of the interface between yolk and the external medium, yields the condition $T_{\rm y} = T_{\rm b} + T_{\rm byi}$.

Similarly, condition (ii) is obtained from the relation arising from Eqs. 11 and 12

$$T_{\rm b} = T_{\rm y}\frac{h_{\rm y}^2 - r_{\rm c}^2 + 2h_{\rm b}h_{\rm y}}{h_{\rm y}^2 + r_{\rm c}^2} + T_{\rm byi}\frac{h_{\rm c}^2 - r_{\rm c}^2 - 2h_{\rm b}h_{\rm c}}{h_{\rm c}^2 + r_{\rm c}^2}. \tag{22}$$

Letting $r_{\rm c} \to 0$ and $h_{\rm b} \to 0$ in the equation above, corresponding to vanishing of the interface between blastoderm and the external medium, yields the condition $T_{\rm b} = T_{\rm y} + T_{\rm byi}$.

Finally, condition (iii) is obtained from the relation

$$T_{\rm byi} = T_{\rm y}\frac{h_{\rm y}^2 - r_{\rm c}^2 + 2h_{\rm c}h_{\rm y}}{h_{\rm y}^2 + r_{\rm c}^2} + T_{\rm b}\frac{h_{\rm b}^2 - r_{\rm c}^2 - 2h_{\rm c}h_{\rm b}}{h_{\rm b}^2 + r_{\rm c}^2} \tag{23}$$

Letting $r_{\rm c} \to 0$ and $h_{\rm c} \to 0$ in the equation above, corresponding to vanishing of the interface between blastoderm and yolk, yields the condition $T_{\rm byi} = T_{\rm b} + T_{\rm y}$.

1.3. **Comparison with experimental data.** Prior to doming, the zebrafish embryo assumes a roughly steady shape during about 10 minutes (Figures 1K-N). Measurements of the embryo shape during this stage are listed in Table S1A. To fit the shape prediction of the previous section to embryo shapes, we impose the value of v_b measured experimentally, solve the predicted shape calculated above, and minimise the following objective function

$$\begin{aligned} S\left(\frac{T_{\rm b}}{T_{\rm y}}, \frac{T_{\rm byi}}{T_{\rm y}}\right) &= (\bar{h}_{\rm y}^t - \bar{h}_{\rm y}^e)^2 + (\bar{h}_{\rm b}^t - \bar{h}_{\rm b}^e)^2 + (\bar{h}_{\rm c}^t - \bar{h}_{\rm c}^e)^2 + (\bar{r}_{\rm c}^t - \bar{r}_{\rm c}^e)^2 \\ &+ \left(1 + \frac{T_{\rm b}}{T_{\rm y}}\cos\varphi^e + \frac{T_{\rm byi}}{T_{\rm y}}\cos\varphi_{\rm y}^e\right)^2 \\ &+ \left(\frac{T_b}{T_{\rm y}}\sin\varphi^e - \frac{T_{\rm byi}}{T_{\rm y}}\sin\varphi_{\rm y}^e\right)^2 \end{aligned} \tag{24}$$

where the t and e indexes respectively stand for theoretical and experimental values. Theoretical values are obtained by solving Eqs. 11 and 12. The last two terms are included in the objective function 24 to ensure that force balance at the contact line is taken into account in the fitting procedure, as the shape of interfaces in the embryo are not perfect portions of sphere. Minimization

of the objective function yields the tension ratio

$$\text{(WT) } T_{\rm b}/T_{\rm y} \simeq 0.94 \pm 0.13 \ , \ T_{\rm byi}/T_{\rm y} \simeq 0.11 \pm 0.24 \tag{25}$$

$$\text{(Depleted) } T_{\rm b}/T_{\rm y} \simeq 0.94 \pm 0.16 \ , \ T_{\rm byi}/T_{\rm y} \simeq 0.09 \pm 0.24 \tag{26}$$

2. Analysis of compression experiments

In this section, we use the surface tension model introduced in section 1 to analyze experiments where the embryo is compressed between two plates and subjected to a constant vertical force $\mathbf{F}$ (Figure S2D). The surface tensions of the upper and lower part of the embryo are determined by quantifying the shape taken by the embryo following compression. Because the embryo has a large enough mass that its shape is affected by gravity, we also take into account here the effect of gravitational forces.

2.1. **Determination of surface tensions.** Following compression between two plates separated by a distance h, a circular surface of contact forms between the embryo and the upper plate, with radius r_u and surface $S_u = \pi r_u^2$. Similarly, a circular region of contact forms with the lower plate with radius r_l and surface $S_l = \pi r_l^2$. The embryo is embedded in Danieau's medium, a fluid with density ρ_0 close to the density of water. As a result the external medium is under a gradient of external pressure $P_0(z) = P_{0u} - \rho_0 g z$, where P_{0u} is the pressure of the fluid in contact with the upper plate. We denote $P_{0l} = P_{0u} - \rho_0 g h$ the pressure of the fluid in contact with the lower plate.

The force exerted by the embryo on the upper plate F and on the lower plate F_l can be written

$$F = (P_u - P_{0u})S_u - T_{\rm b} 2\pi r_u \sin\varphi_u, \tag{27}$$

$$F_l = (P_l - P_{0l})S_l - T_{\rm y} 2\pi r_l \sin\varphi_l, \tag{28}$$

where we denote here P_u the pressure in the blastoderm near the upper plate and P_l the pressure in the yolk near the lower plate.

The pressures within the yolk and the blastoderm are related to the surface tensions $T_{\rm b}$ and $T_{\rm y}$ by the law of Laplace applied to a section of the surface near the plane of contact:

$$P_u - P_{0u} = \frac{2T_{\rm b}}{R_u}, \tag{29}$$

$$P_l - P_{0l} = \frac{2T_{\rm y}}{R_l}, \tag{30}$$

where $1/R_u$ and $1/R_l$ denotes the mean curvature of the surface of the embryo near the planes of contact. For simplicity and because changes of curvature across the surface of the blastoderm or

the yolk can not be resolved experimentally with enough precision, we calculated the curvatures from segmentations of the entire blastoderm and yolk surfaces.

Using Eqs. 27-30 and eliminating the pressures, one finds the two relations

$$\frac{2T_{\mathrm{b}}}{R_u} = \frac{F}{\pi r_u^2(1 - R_u/r_u \sin\varphi_u)}, \tag{31}$$

$$\frac{2T_{\mathrm{y}}}{R_l} = \frac{F_l}{\pi r_l^2(1 - R_l/r_l \sin\varphi_l)}. \tag{32}$$

We now relate the force exerted on the lower plate F_l to the force exerted on the upper plate F. Denoting ρ the mass density of the embryo, Archimedes' principle states that

$$F_l - F = (\rho - \rho_0)gV \tag{33}$$

where V is the volume of the embryo.

Combining these equations, we obtain finally

$$\frac{2T_{\mathrm{b}}}{R_u} = \frac{F}{\pi r_u^2(1 - R_u/r_u \sin\varphi_u)} \tag{34}$$

$$\frac{2T_{\mathrm{y}}}{R_l} = \frac{F + (\rho - \rho_0)gV}{\pi r_l^2(1 - R_l/r_l \sin\varphi_l)} \tag{35}$$

By measuring the force F, the radii of contact r_u and r_l, the contact angles φ_u and φ_l, the density of the embryo relative to the medium ρ/ρ_0, the mean radii of curvature of the yolk and blastoderm R_u and R_l, Eqs. 34 and 35 yield the surface tensions T_{b} and T_{y}.

2.2. **Determination of the mass of the embryo.** To measure the mass of the embryo $m = \rho V$, we performed sinking experiments and recorded the steady-state sinking velocity of the embryo. The Reynolds number is defined as

$$\mathrm{Re} = \frac{2\rho_0 R U}{\eta_0} \tag{36}$$

with ρ_0 the medium density, η_0 the medium viscosity, R the radius of the embryo and U its sinking velocity. Using experimental measurements listed in Table S1B, we find that the Reynolds numbers are respectively $\mathrm{Re} = 9.56$ for WT embryo and $\mathrm{Re} = 9.21$ for deep cell depleted embryos. As a result, the sinking of embryos does not occur at low Reynolds number, and the sinking velocity can be related to the mass density of the embryo through

$$U = \sqrt{\frac{8gR}{3C_d}\frac{\rho - \rho_0}{\rho_0}} \tag{37}$$

with C_d the drag coefficient [Clift et al. (2005)]. For a sphere, the drag coefficient is a function of the Reynolds number; using the approximate formula of [Clift et al. (2005)] we find respectively

$C_d \simeq 4.39$ for WT embryos and $C_d \simeq 4.5$ for deep-cell depleted embryos. Using Eq. 37, we then obtain

$$\rho_{\text{WT}} = 1055.59 \text{ kg/m}^3 \quad (38)$$

$$\rho_{\text{Depleted}} = 1053.44 \text{ kg/m}^3 \quad (39)$$

3. Fluid dynamic description of zebrafish doming

In this section, we propose a dynamic description of zebrafish doming where the blastoderm and EVL are described as active fluids (Figures 2 and S2E).

3.1. General equations.

3.1.1. *Blastoderm flow and stress tensors.* We label a position in the blastoderm $\mathbf{X}(x^1, x^2, x^3)$ by the curvilinear coordinates x^1, x^2, x^3 (Figure S2E). In the following, 3D coordinates are denoted by greek indices, lower (resp. upper) indices correspond to covariant (resp. contravariant) coordinates, and Einstein summation is implied. The associated local 3D basis is denoted $\mathbf{e}_\alpha = \partial_\alpha \mathbf{X}$. We denote the associated metric as $g_{\alpha\beta} = \mathbf{e}_\alpha \cdot \mathbf{e}_\beta$ and the metric determinant as $g = \det g_{\alpha\beta}$. In practice, in simulations, we use cylindrical coordinates r, ϕ, z around the animal-vegetal axis of the embryo.

The blastoderm is described as an active, incompressible fluid with velocity field $\mathbf{v} = v^\alpha \mathbf{e}_\alpha$. The incompressibility condition imposes that

$$(\partial_\alpha \mathbf{v}) \cdot \mathbf{e}^\alpha = 0 \quad (40)$$

The constitutive equation for the stress in the fluid $\sigma_{\alpha\beta}$ is given by

$$\sigma_{\alpha\beta} = 2\eta v_{\alpha\beta} - P g_{\alpha\beta} + \zeta_{\alpha\beta} \quad (41)$$

where η is the shear viscosity of the fluid, $v_{\alpha\beta} = \frac{1}{2}((\partial_\alpha \mathbf{v}) \cdot \mathbf{e}_\beta + (\partial_\beta \mathbf{v}) \cdot \mathbf{e}_\alpha)$ is the symmetric gradient of velocity, the pressure P ensures incompressibility of the fluid, and $\zeta_{\alpha\beta}$ is a symmetric traceless tensor corresponding to an active internal anisotropic stress. The form of $\zeta_{\alpha\beta}$ depends on internal polarised cellular processes driving autonomous tissue deformations. In the following we assume that the active stress is oriented radially, away from the centre of the embryo:

$$\zeta_{\alpha\beta} = \zeta \left(n_\alpha n_\beta - \frac{1}{3} g_{\alpha\beta} \right), \quad (42)$$

with $\mathbf{n}$ a unit vector characterising the orientation of the anisotropic active stress. Using the cylindrical coordinates r, z, ϕ around the animal-vegetal axis, we choose the orientation (Figure

S2E):

$$\mathbf{n} = \frac{1}{\sqrt{r^2 + (z - z_0)^2}} \left(r\mathbf{e}_r + (z - z_0)\mathbf{e}_z \right). \tag{43}$$

where $z_0 = (z_a + z_v)/2$ is a position along the animal-vegetal axis, with z_a and z_v the positions of the animal and vegetal poles. Because the embryo has roughly the shape of a sphere, this choice ensures that the vector $\mathbf{n}$ is approximately orthogonal to the surface of the embryo.

Force balance in the blastoderm reads

$$\partial_\alpha(\sqrt{g}\sigma^{\alpha\beta}\mathbf{e}_\beta) = 0. \tag{44}$$

Plugging the constitutive equation 41 in the force balance equation 44 yields an equation for the velocity field $\mathbf{v}$. To solve this equation, boundary conditions for the stress at the surface of the blastoderm must be specified. In the next section, we describe the constitutive equations for the surface tension of the blastoderm.

3.1.2. *Blastoderm surface flow and tension tensor.* We now give constitutive equations for the flows at the surface of the blastoderm, in the EVL. We assume that the normal velocities of the EVL and blastoderm at the surface coincide, but the EVL and blastoderm can have different tangential velocities. We express covariant constitutive equations using the framework of differential geometry for surfaces [Deserno (2004)].

The surface $\mathcal{S}$ enclosing the blastoderm is labelled by the vector $\bar{\mathbf{X}}(s^1, s^2)$, where s^1 and s^2 are two coordinates going along the surface. In the following, coordinates on the surface are denoted by latin indices i, j, k, l, lower (resp. upper) indices correspond to covariant (resp. contravariant) coordinates, and Einstein summation is implied. The tangent and normal vectors to the surface are defined by

$$\bar{\mathbf{e}}_i = \partial_i \bar{\mathbf{X}}, \tag{45}$$

$$\bar{\mathbf{n}} = \frac{\bar{\mathbf{e}}_1 \times \bar{\mathbf{e}}_2}{|\bar{\mathbf{e}}_1 \times \bar{\mathbf{e}}_2|}. \tag{46}$$

Coordinates are chosen such that the normal vector points outward the blastoderm. The curvature tensor C_{ij} can then be obtained from $C_{ij} = \bar{\mathbf{e}}_i \cdot \partial_j \bar{\mathbf{n}}$, the metric tensor reads $\bar{g}_{ij} = \bar{\mathbf{e}}_i \cdot \bar{\mathbf{e}}_j$ and the metric determinant is denoted by $\bar{g} = \det \bar{g}_{ij}$. We denote the covariant derivative on the surface $\bar{\nabla}_i$, defined by its application on a tangent vector $\mathbf{v}$ through $(\partial_i \mathbf{v}) \cdot \bar{\mathbf{e}}^j = \bar{\nabla}_i v^j$.

The surface is flowing according to a velocity field $\bar{\mathbf{v}}$ (Figure S2E), which can be separated in a tangential and normal part:

$$\bar{\mathbf{v}} = \bar{v}^i \bar{\mathbf{e}}_i + \bar{v}^n \bar{\mathbf{n}}. \tag{47}$$

For convenience we also introduce the difference between the bulk surface flow and the EVL flow:

$$\mathbf{u} = \bar{\mathbf{v}} - \mathbf{v}. \tag{48}$$

Because the normal velocities of the blastoderm and its surface coincide, $\mathbf{u}$ is a tangential vector field. We also impose that the blastoderm and EVL move at the same velocity at the contact line; therefore

$$\mathbf{u}\big|_{\mathcal{C}} = 0. \tag{49}$$

Two surfaces enclose the blastoderm: the EVL surface $\mathcal{S}_b$ with surface tension tensor $\mathbf{t}_{\mathrm{b}}^i = t_{\mathrm{b}}^{ij}\mathbf{e}_j$ and the blastoderm-yolk interface $\mathcal{S}_{\mathrm{byi}}$ with uniform isotropic surface tension $\mathbf{t}_{\mathrm{byi}}^i = T_{\mathrm{byi}}\mathbf{e}^i$ (Figure S2E). We assume here that the viscosity of the BYI is negligible. The surface tension tensor of the EVL is given by the constitutive equation

$$\begin{aligned} t_{\mathrm{b}}^{ij} &= t_{\mathrm{b}}\bar{g}^{ij}, \\ t_{\mathrm{b}} &= T_{\mathrm{b}} + \bar{\eta}(\partial_j \bar{\mathbf{v}}) \cdot \bar{\mathbf{e}}^j. \end{aligned} \tag{50}$$

where we have introduced the active tension of the blastoderm surface T_b, and the surface viscosity of the EVL $\bar{\eta}$. The force balance equation of the EVL and BYI read, taking into account the force exerted by the blastoderm as well as the external fluid pressure P_0 and P_y:

$$\bar{\nabla}_i \mathbf{t}_{\mathrm{b}}^i + \mathbf{f} - P_0 \bar{\mathbf{n}} = 0, \tag{51}$$

$$\bar{\nabla}_i \mathbf{t}_{\mathrm{byi}}^i + \mathbf{f} - P_{\mathrm{y}} \bar{\mathbf{n}} = 0, \tag{52}$$

where we have introduced the force $\mathbf{f}$ exerted by the fluid blastoderm on the surface. The force $\mathbf{f}$ is related to the bulk blastoderm stress tensor according to

$$f^\alpha = -\sigma^{\beta\alpha}\bar{n}_\beta. \tag{53}$$

We assume that a friction force acts at the boundary between the volume and the surface with friction coefficient ξ at the EVL interface, and we assume that there is no friction acting at the BYI. Decomposing the force exerted by the blastoderm on the EVL into its parallel and normal contributions $\mathbf{f} = f^i\mathbf{e}_i + f_n\bar{\mathbf{n}}$ and using the force balance equation 51,

$$f_n = P_0 + t_b C_k{}^k, \tag{54}$$

$$f_i = \xi(v_i - \bar{v}_i) = -\xi u_i = -\bar{\nabla}_i t_b. \tag{55}$$

In the following, we set the external reference pressure P_0 to be equal to 0. Similarly, one obtains at the BYI

$$f_n = P_{\mathrm{y}} + T_{\mathrm{byi}} C_k{}^k, \tag{56}$$

$$f_i = 0, \tag{57}$$

where we have chosen here to neglect the friction of the blastoderm against the BYI.
Finally, the surfaces $\mathcal{S}_{\mathrm{b}}$, $\mathcal{S}_{\mathrm{byi}}$ and $\mathcal{S}_{\mathrm{y}}$ meet on a contour line denoted $\mathcal{C}$ (Figure S2E). Force balance on the contour $\mathcal{C}$ implies

$$\mathbf{t}_{\mathrm{b}}^{i} \boldsymbol{\nu}_{\mathrm{b,i}} = -T_{\mathrm{byi}} \boldsymbol{\nu}_{\mathrm{byi}} - T_{\mathrm{y}} \boldsymbol{\nu}_{\mathrm{y}}, \text{ on the contour } \mathcal{C} \tag{58}$$

where $\boldsymbol{\nu}_{\mathrm{b}}$, $\boldsymbol{\nu}_{\mathrm{byi}}$ and $\boldsymbol{\nu}_{\mathrm{y}}$ are the vectors tangent to the surface $\mathcal{S}_{\mathrm{b}}$, $\mathcal{S}_{\mathrm{byi}}$ and $\mathcal{S}_{\mathrm{y}}$ respectively, and are all normal to the contour $\mathcal{C}$ (Figures S2C and E).

3.2. **Shape of the yolk.** The viscosity of the yolk is taken to be negligible compared to other dissipative processes. The shape of the yolk surface is therefore a spherical cap with radius of curvature R_{y}. Volume conservation of the yolk imposes

$$V_{\mathrm{y}} = \frac{\pi}{6} h_{\mathrm{y}} (3 r_{\mathrm{c}}^2 + h_{\mathrm{y}}^2) + \int_{\mathcal{S}_0} dS z, \tag{59}$$

where the volume of the yolk has been decomposed as the volume of a spherical cap and the volume contained between $\mathcal{S}_{\mathrm{byi}}$ and the planar region $\mathcal{S}_0$ perpendicular to the animal-vegetal axis ($z = 0$), contained within the contact line. Eq. 59 can be solved for h_{y}, and yields then the radius of curvature R_{y} through the relation $R_{\mathrm{y}} = (r_{\mathrm{c}}^2 + h_{\mathrm{y}}^2)/2h_{\mathrm{y}}$. The yolk pressure is then imposed by the law of Laplace

$$P_{\mathrm{y}} = \frac{2T_{\mathrm{y}}}{R_{\mathrm{y}}}. \tag{60}$$

3.3. **Parameter list.** Eqs. 44, 51, 56 and 58-60 form a system of equations which can be solved for the velocity of the blastoderm, the velocity of the EVL and the shape of the yolk, once surface tensions, active stresses and viscosities are specified. The corresponding physical parameters are listed in Table S1C. The surface tension of the yolk T_{y} is determined from compression experiments described in section 2.

3.4. **Finite element simulations.** We now describe the Galerkin method [Zienkiewicz et al. (1977)] we used to perform finite element simulations for the blastoderm bulk and surface flows, according to the equations described above.

3.4.1. *Bulk flow.* To ensure the force balance condition in bulk Eq. 44, we introduce a test vector field $\mathbf{w}$ and write the weak formulation as an integral over the volume $\mathcal{V}$:

$$\int_{\mathcal{V}} dx^1 dx^2 dx^3 \mathbf{w} \cdot \partial_\alpha(\sqrt{g}\sigma^{\alpha\beta}\mathbf{e}_\beta) = 0. \tag{61}$$

Performing an integration by part, Eq. 61 can be rewritten

$$-\int_{\mathcal{V}} dV(\partial_\alpha \mathbf{w}) \cdot (\sigma^{\alpha\beta}\mathbf{e}_\beta) + \int_{\mathcal{S}} dS\bar{n}_\alpha w_\beta \sigma^{\alpha\beta} = 0. \tag{62}$$

where we have introduced the volume element $dV = \sqrt{g}dx^1dx^2dx^3$, the surface element $dS = \sqrt{\bar{g}}ds^1ds^2$, and $\mathcal{S}$ denotes the union of $\mathcal{S}_{\rm b}$ and $\mathcal{S}_{\rm byi}$. Using the constitutive equation 41 for the stress tensor $\sigma_{\alpha\beta}$, the definition of the boundary force $\mathbf{f}$ given in Eq. 53, and the force balance equation on the surface 51, we obtain

$$\begin{aligned}-\int_{\mathcal{V}} dV 2\eta(\partial_\alpha \mathbf{w}) \cdot \mathbf{e}_\beta v^{\alpha\beta} + \int_{\mathcal{V}} dVP(\partial_\alpha \mathbf{w}) \cdot \mathbf{e}^\alpha - \int_{\mathcal{V}} dV(\partial_\alpha \mathbf{w}) \cdot \mathbf{e}_\beta \zeta^{\alpha\beta} \\ + \int_{\mathcal{S}_{\rm b}} dS\mathbf{w} \cdot (\bar{\nabla}_i \mathbf{t}^i_{\rm b} - P_0\bar{\mathbf{n}}) + \int_{\mathcal{S}_{\rm byi}} dS\mathbf{w} \cdot (\bar{\nabla}_i \mathbf{t}^i_{\rm byi} - P_{\rm y}\bar{\mathbf{n}}) = 0.\end{aligned} \tag{63}$$

Performing an integration by part of the surface terms and setting the reference pressure $P_0 = 0$, one obtains

$$\begin{aligned}-\int_{\mathcal{V}} dV 2\eta(\partial_\alpha \mathbf{w}) \cdot \mathbf{e}_\beta v^{\alpha\beta} + \int_{\mathcal{V}} dVP(\partial_\alpha \mathbf{w}) \cdot \mathbf{e}^\alpha - \int_{\mathcal{V}} dV(\partial_\alpha \mathbf{w}) \cdot \mathbf{e}_\beta \zeta^{\alpha\beta} \\ - \int_{\mathcal{S}_{\rm b}} dS(\partial_i \mathbf{w}) \cdot \mathbf{t}^i_{\rm b} - \int_{\mathcal{S}_{\rm byi}} dS(\partial_i \mathbf{w}) \cdot \mathbf{t}^i_{\rm byi} - \int_{\mathcal{S}_{\rm byi}} dSP_{\rm y}\mathbf{w} \cdot \bar{\mathbf{n}} \\ + \int_{\mathcal{C}} dl\nu_{\rm b,i}\mathbf{w} \cdot \mathbf{t}^i_{\rm b} + \int_{\mathcal{C}} dl\nu_{\rm byi,i}\mathbf{w} \cdot \mathbf{t}^i_{\rm byi} = 0,\end{aligned} \tag{64}$$

where we have introduced the infinitesimal line element dl on the contour $\mathcal{C}$. Using the constitutive equations 50 and the force balance equation 58 on the contour $\mathcal{C}$,

$$\begin{aligned}-\int_{\mathcal{V}} dV 2\eta(\partial_\alpha \mathbf{w}) \cdot \mathbf{e}_\beta v^{\alpha\beta} + \int_{\mathcal{V}} dVP(\partial_\alpha \mathbf{w}) \cdot \mathbf{e}^\alpha - \int_{\mathcal{V}} dV(\partial_\alpha \mathbf{w}) \cdot \mathbf{e}_\beta \zeta^{\alpha\beta} \\ - \int_{\mathcal{S}_{\rm b}} dS(\partial_i \mathbf{w}) \cdot \bar{\mathbf{e}}^i(T_{\rm b} + \bar{\eta}(\partial_j \bar{\mathbf{v}}) \cdot \bar{\mathbf{e}}^j) - T_{\rm byi}\int_{\mathcal{S}_{\rm byi}} dS(\partial_i \mathbf{w}) \cdot \bar{\mathbf{e}}^i \\ - \int_{\mathcal{S}_{\rm byi}} dSP_{\rm y}\mathbf{w} \cdot \bar{\mathbf{n}} - T_{\rm y}\int_{\mathcal{C}} dl\mathbf{w} \cdot \boldsymbol{\nu}_{\rm y} = 0.\end{aligned} \tag{65}$$

The velocity, pressure, active stress, surface tension fields are decomposed on a set of basis function $\{\psi^a(\mathbf{x})\}$, $\{\chi^b(\mathbf{x})\}$, $\tilde{\chi}^a(\mathbf{x})\}$ and $\{\chi_s^c(\mathbf{x})\}$:

$$\mathbf{v}(\mathbf{x}) = \sum_a v^{a,\alpha}\mathbf{e}_\alpha\psi^a(\mathbf{x}) \tag{66}$$

$$\mathbf{w}(\mathbf{x}) = \sum_a w^{a,\alpha}\mathbf{e}_\alpha\psi^a(\mathbf{x}) \tag{67}$$

$$\mathbf{u}(\mathbf{x}) = \sum_a u^{a,j}\bar{\mathbf{e}}_j\psi^a(\mathbf{x}) \tag{68}$$

$$P(\mathbf{x}) = \sum_b P^b\chi^b(\mathbf{x}) \tag{69}$$

$$\zeta^{\alpha\beta}(\mathbf{x}) = \sum_a \zeta^{a,\alpha\beta}\tilde{\chi}^a(\mathbf{x}) \tag{70}$$

$$T_{\mathrm{b}}(\mathbf{x}) = \sum_c T_{\mathrm{b}}^c\chi_s^c(\mathbf{x}). \tag{71}$$

Note that the surface flow $\mathbf{u}$ and surface tension T_b only take non-zero values on elements at the surface of the blastoderm. Using this decomposition, eq. 65 takes the form of a matrix equation

$$w^{a,\alpha}\Big[-M^1_{(a,\alpha),(a',\beta)}v^{a',\beta} + M^2_{(a,\alpha),b}P^b - M^3_{(a,\alpha),(a,\beta,\gamma)}\zeta^{a,\beta\gamma}$$

$$-M^4_{(a,\alpha),c}T^c_{\mathrm{b}} - M^5_{(a,\alpha),(a',\beta)}v^{a',\beta} - \bar{M}^2_{(a',i),(a,\alpha)}u^{a',i} - M^6_{(a,\alpha)}T_{\mathrm{byi}} - M^7_{(a,\alpha)}P_{\mathrm{y}} - M^8_{(a,\alpha)}T_{\mathrm{y}}\Big] = 0, \tag{72}$$

with the coefficients

$$M^1_{(a,\alpha),(a',\beta)} = \int_{\mathcal{V}} dV\eta\left(\partial_\gamma(\psi^a\mathbf{e}_\alpha)\cdot\mathbf{e}_\delta\right)\Big(\partial^\gamma(\psi^{a'}\mathbf{e}_\beta)\cdot\mathbf{e}^\delta + \partial^\delta(\psi^{a'}\mathbf{e}_\beta)\cdot\mathbf{e}^\gamma\Big) \tag{73}$$

$$M^2_{(a,\alpha),b} = \int_{\mathcal{V}} dV\partial_\gamma(\psi^a\mathbf{e}_\alpha)\cdot\mathbf{e}^\gamma\chi^b \tag{74}$$

$$M^3_{(a,\alpha),(a,\beta,\gamma)} = \int_{\mathcal{V}} dV\partial_\beta(\psi^a\mathbf{e}_\alpha)\cdot\mathbf{e}_\gamma\tilde{\chi}^a \tag{75}$$

$$M^4_{(a,\alpha),c} = \int_{\mathcal{S}_{\mathrm{b}}} dS\partial_i(\psi^a\mathbf{e}_\alpha)\cdot\bar{\mathbf{e}}^i\chi_s^c \tag{76}$$

$$M^5_{(a,\alpha),(a',\beta)} = \int_{\mathcal{S}_{\mathrm{b}}} dS\bar{\eta}\partial_i(\psi^a\mathbf{e}_\alpha)\cdot\bar{\mathbf{e}}^i\partial_j(\psi^{a'}\mathbf{e}_\beta)\cdot\bar{\mathbf{e}}^j \tag{77}$$

$$M^6_{(a,\alpha)} = \int_{\mathcal{S}_{\mathrm{byi}}} dS\partial_i(\psi^a\mathbf{e}_\alpha)\cdot\bar{\mathbf{e}}^i \tag{78}$$

$$M^7_{(a,\alpha)} = \int_{\mathcal{S}_{\mathrm{byi}}} dS\psi^a\mathbf{e}_\alpha\cdot\bar{\mathbf{n}} \tag{79}$$

$$M^8_{(a,\alpha)} = \int_{\mathcal{C}} dl\psi^a\mathbf{e}_\alpha\cdot\boldsymbol{\nu}_y. \tag{80}$$

and $\bar{M}^2_{(a',i),(a,\alpha)}$ is defined in Eq. 91. Because Eq. 3.4.1 must be valid for all test functions $\mathbf{w}$, the velocity field obeys the matrix equation

$$\left(M^1_{(a,\alpha),(a',\beta)} + M^5_{(a,\alpha),(a',\beta)}\right) v^{a',\beta} + \bar{M}^2_{(a',i),(a,\alpha)} u^{a',i} = M^2_{(a,\alpha),b} P^b$$
$$-M^3_{(a,\alpha),(b,\beta,\gamma)} \zeta^{b,\beta\gamma} - M^4_{(a,\alpha),a'} T^{a'}_{\mathrm{b}} - M^6_{(a,\alpha)} T_{\mathrm{byi}} - M^7_{(a,\alpha)} P_{\mathrm{y}} - M^8_{(a,\alpha)} T_{\mathrm{y}}. \quad (81)$$

To ensure incompressibility (Eq. 40), we write the weak form formulation:

$$\int_{\mathcal{V}} dV w_s (\partial_\alpha \mathbf{v}) \cdot \mathbf{e}^\alpha = 0. \quad (82)$$

Decomposing the test function w_s on the basis function χ^b,

$$w_s(\mathbf{x}) = \sum_b w_s^b \chi^b(\mathbf{x}), \quad (83)$$

Eq. 82 can be rewritten

$$M^2_{(a,\alpha),b} v^{a,\alpha} = 0 \quad (84)$$

where $M^2_{(a,\alpha),b}$ is defined in Eq. 74.

3.4.2. *Surface flow.* We now derive an equation for the flow on the surface $\mathcal{S}_{\mathrm{b}}$. We introduce a test vector field $\bar{\mathbf{w}}$ tangent to the surface, and write the weak formulation as an integral over the surface $\mathcal{S}_{\mathrm{b}}$:

$$\int_{\mathcal{S}_{\mathrm{b}}} dS \bar{\mathbf{w}} \cdot (\bar{\nabla}_i \mathbf{t}^i_{\mathrm{b}} + \mathbf{f}) = 0. \quad (85)$$

where $dS = \sqrt{\bar{g}} ds^1 ds^2$ is the surface element on $\mathcal{S}_{\mathrm{b}}$. Integration by part then yields the modified equation

$$-\int_{\mathcal{S}_{\mathrm{b}}} dS (\partial_i \bar{\mathbf{w}}) \cdot \mathbf{t}^i_{\mathrm{b}} + \int_{\mathcal{S}_{\mathrm{b}}} dS \bar{\mathbf{w}} \cdot \mathbf{f} + \int_{\mathcal{C}} dl \nu_{\mathrm{b,i}} \bar{\mathbf{w}} \cdot \mathbf{t}^i_{\mathrm{b}} = 0 \quad (86)$$

where $\boldsymbol{\nu}_{\mathrm{b}}$ is the unit vector tangent to $\mathcal{S}_{\mathrm{b}}$ and normal to the contour $\mathcal{C}$. Using the constitutive equation for the surface tension 50, one obtains

$$-\int_{\mathcal{S}_{\mathrm{b}}} dS \bar{\eta} ((\partial_i \bar{\mathbf{w}}) \cdot \bar{\mathbf{e}}^i)((\partial_j \mathbf{u}) \cdot \bar{\mathbf{e}}^j) - \int_{\mathcal{S}_{\mathrm{b}}} dS \bar{\eta} ((\partial_i \bar{\mathbf{w}}) \cdot \bar{\mathbf{e}}^i)((\partial_j \mathbf{v}) \cdot \bar{\mathbf{e}}^j)$$
$$-\int_{\mathcal{S}_{\mathrm{b}}} dS T_{\mathrm{b}} (\partial_i \bar{\mathbf{w}}) \cdot \bar{\mathbf{e}}^i - \int_{\mathcal{S}_{\mathrm{b}}} dS \xi \bar{w}^i u_i + \int_{\mathcal{C}} dl \bar{\mathbf{w}} \cdot (T^c_b \boldsymbol{\nu}_b) = 0 \quad (87)$$

where T^c_b is the surface tension at the contact point. Discretizing the fields $\mathbf{u}$ and T_b on the surface (Eq. 68 and 71) as well as the field $\bar{\mathbf{w}}$:

$$\bar{\mathbf{w}}(\mathbf{x}) = \sum_a \bar{w}^{a,i} \bar{\mathbf{e}}_i \psi^a(\mathbf{x}), \quad (88)$$

Eq. 87 takes the form

$$\bar{w}^{a,i}(-\bar{M}^1_{(a,i),(a',j)}u^{a',j} - \bar{M}^2_{(a,i),(a',\alpha)}v^{a',\alpha} - \bar{M}^3_{(a,i),(a',j)}u^{a',j} - \bar{M}^4_{(a,i),a'}T_b^{a'} + \bar{M}^5_{a,i}) = 0, \quad (89)$$

with the following coefficients

$$\bar{M}^1_{(a,i),(a',j)} = \int_{\mathcal{S}_b} dS\bar{\eta}((\partial_k(\psi^a\bar{\mathbf{e}}_i))\cdot\bar{\mathbf{e}}^k)((\partial_l(\psi^{a'}\bar{\mathbf{e}}_j))\cdot\bar{\mathbf{e}}^l) \quad (90)$$

$$\bar{M}^2_{(a,i),(a',\alpha)} = \int_{\mathcal{S}_b} dS\bar{\eta}((\partial_k(\psi^a\bar{\mathbf{e}}_i))\cdot\bar{\mathbf{e}}^k)((\partial_l(\psi^{a'}\mathbf{e}_\alpha))\cdot\bar{\mathbf{e}}^l) \quad (91)$$

$$\bar{M}^3_{(a,i),(a',j)} = \int_{\mathcal{S}_b} dS\xi\psi^a\psi^{a'}\delta_{ij} \quad (92)$$

$$\bar{M}^4_{(a,i),(a')} = \int_{\mathcal{S}_b} dS((\partial_k(\psi^a\bar{\mathbf{e}}_i))\cdot\bar{\mathbf{e}}^k\psi^{a'} \quad (93)$$

$$\bar{M}^5_{(a,i)} = \int_{\mathcal{C}} dl\psi^a\bar{\mathbf{e}}_i\cdot(\boldsymbol{\nu}_\mathrm{b}T_b^c) \quad (94)$$

Eq. 89 must be valid for all test functions $\bar{\mathbf{w}}$ and can therefore be rewritten

$$(\bar{M}^1_{(a,i),(a',j)} + \bar{M}^3_{(a,i),(a',j)})u^{a',j} + \bar{M}^2_{(a,i),(a',j)}v^{a',j} = -\bar{M}^4_{(a,i),a'}T_\mathrm{b}^{a'} + \bar{M}^5_{a,i}. \quad (95)$$

T_b^c can be seen as a Lagrange multiplier enforcing condition 49.

3.4.3. *Flow near the contact line.* Stokes flow solutions near a corner can exhibit divergences of the pressure and gradient of flow (Moffatt (1964), De Gennes (1985), Huh and Scriven (1971)). The continuum description of tissue flow we propose here however does not apply on length scales smaller than a cell diameter $l_c \sim 15-20\mu$m, effectively setting a cut-off near the contact line. In our finite-element simulations, this is ensured by the finite size of the elements. As a result, the force balance equation at the contact line becomes

$$\mathbf{t}_\mathrm{b}^i\boldsymbol{\nu}_\mathrm{b,i} + T_\mathrm{byi}\boldsymbol{\nu}_\mathrm{byi} + T_\mathrm{y}\boldsymbol{\nu}_\mathrm{y} = \mathbf{t}_D, \quad (96)$$

with $\mathbf{t}_D$ a tension dependent on l_c. This correction arises from forces acting on a length l_c and contributing a force near the contact line.

3.5. **Implementation.** Eqs. 81, 84 and 95 form a system of linear equations which can be solved for the vectors of bulk velocities $v^{a,\alpha}$, relative surface velocities $u^{a,j}$ and bulk pressure P^b.

To implement the finite element simulation, we choose a mesh consisting of $N = 384$ quadrilaterals made from 8 triangles with a common vertex. The shape functions ψ^a for the velocity field are chosen as linear functions on triangles, the shape functions χ^b for the pressure are taken constant on quadrilaterals, and the shape functions $\tilde{\chi}^a$ are taken as constant on triangles. The

shape function for the surface tension T_b, χ_s^c, are taken constant on surface elements. We use cylindrical coordinates (r, ϕ, z) and assume axisymmetry, such that the stresses and velocity fields do not depend on ϕ. The basis vectors $\mathbf{e}_\alpha$ are therefore given by

$$\mathbf{e}_r = \cos\phi \mathbf{e}_x + \sin\phi \mathbf{e}_y \ , \ \mathbf{e}_\phi = r(-\sin\phi \mathbf{e}_x + \cos\phi \mathbf{e}_y) \ , \ \mathbf{e}_z \tag{97}$$

with $\mathbf{e}_x$, $\mathbf{e}_y$, $\mathbf{e}_z$ the unit vectors of a right-handed cartesian coordinate system (Figure S2E). On the surface, we take coordinates (s, ϕ) such that point on the surface is given by

$$\bar{\mathbf{X}}(s, \phi) = r(s)\cos\phi \mathbf{e}_x + r(s)\sin\phi \mathbf{e}_y + z(s)\mathbf{e}_z. \tag{98}$$

with $z(s)$ and $r(s)$ two functions characterising the shape of the surface. Because of axisymmetry, the surface velocities only have one non-zero component, $\bar{v}_s$, u_s.

The overall velocity of the embryo is specified by external forces that we do not take into account here. We therefore set the velocity component v_z of the vertex with maximum z to 0. In addition, radial velocities $v_r = 0$ of vertices located on the axis of symmetry are set to 0. Eq. 49 is enforced by imposing that the velocity u_s vanishes at the vertex located on the contact line.

At each time step t, the components of the bulk and surface velocity fields $\mathbf{v}$ and $\mathbf{u}$ are calculated by inverting eqs. 81 and 95. The mesh is then updated at $t + dt$ by moving the mesh points according to the bulk velocity field $\mathbf{v}$. In addition, the radius of the curvature of the yolk R_y is updated to take into account the volume conservation of the yolk, Eq. 59. This results in a change of yolk pressure according to Eq. 60.

Doming is simulated in WT embryos by assuming that at $t = 0$, the EVL surface tension is reduced by a constant factor (Table S1C) and that the active stress ζ, which vanishes for $t < 0$, takes a constant non zero value for $t > 0$. The computed pressure field is shown in Figures S4I-I'. Deep-cell depleted and *pky* embryos are simulated similarly with different parameters (Table S1C).

To simulate grafting experiments described in Figure 5, we perform simulations with a profile of surface tension $T_\mathrm{b}(s)$:

$$s < s_r : T_\mathrm{b}(s) = T_\mathrm{b}^r \tag{99}$$

$$s > s_r : T_\mathrm{b}(s) = T_\mathrm{b}^0 \tag{100}$$

with T_b^0 the initial surface tension before rescue, and T_b^r the surface tension in the grafted patch of EVL. s_r is updated in time according to the surface flow.

When considering a corresponding rescue of active stress within the blastoderm(scenario 3, Table S1D), we define the radius $r_r = r(s_r)$ with $r(s)$ the radial profile of the EVL prior to doming

defined in Eq.98, and introduce a spatial profile of active stress $\zeta(r, z)$:

$$r < r_r : \zeta(r, z) = \zeta^r \tag{101}$$

$$r > r_r : \zeta(r, z) = \zeta^0 \tag{102}$$

with ζ^0 the blastoderm active stress before rescue, and ζ^r the blastoderm active stress in the region below the grafted EVL patch. We take a fixed value of r_r for simplicity.

To test the effect of a friction force between the blastoderm and EVL (Figure 6), we have to specify a physically realistic value of friction coefficient ξ in simulations. To have a significant effect on blastoderm flows, the friction coefficient must be large enough, $\eta/\xi < R$. With values for WT embryos (Table S1C), this imposes $\xi > 2.6$ Pa.s/μm. We also note that EVL flows are spread over the entire EVL [Behrndt et al., 2012], implying that the hydrodynamic length $l = \sqrt{\bar{\eta}/\xi}$ can not be much smaller than the radius of the embryo R. Taking this into account, we performed simulations choosing a friction coefficient in the upper range of possible values, $\xi = 150$ Pa.s/μm, corresponding to a hydrodynamic length $l = 72\mu$m, of the same order of magnitude than the radius of the embryo.

3.6. **Simulation of fusion experiment.** In order to determine the viscosity of the blastoderm, η_b, we implemented finite elements simulations reproducing the fusion experiment of two WT explants, described in "Methods details - Embryo compression and relaxation experiments". We first relaxed a sphere with fixed volume V and fixed the equatorial radius r_e to a value of $r_e/V^{1/3}$ set by experimental observations of initial conditions of fusion. We then relaxed the constraint on the radius and calculated the pressure and velocity field as given by Eq. 81 and 95, without the terms describing the yolk and blastoderm-yolk interface, and without active stress. The mesh was then deformed over time according to the velocity field. The surface tension of the explant was set according to values measured from compression experiments ($T_b \simeq 94$pN/μm, the surface tension of explants was found to be different from the surface tension of embryos). We then adjusted the viscosity η and kept the ratio of $\bar{\eta}/\eta$ equal to the WT value given in Table S1C. We computed the length of the long axis of the two explants and compared the result to experimental measurement (Figure S3I).

3.7. **Simulation of compression-release experiment.** In order to determine the viscosity of the yolk, η_y, we implemented finite elements simulations reproducing the compression-release experiment on one yolk explant as explained in "Methods details - Embryo compression and relaxation experiments".

Using the measurement of the initial distance between the two plates, we initialised the mesh and relaxed it by keeping its volume constant and constraining the vertices in contact with the plates (compression phase). We then removed the constraint and let the entire mesh relax to its equilibrium shape (release phase), setting the viscosity of the fluid represented by the mesh to η_y. We set the yolk surface tension as in the experimental measurements obtained from the same compression experiments ($T_y = 146$ pN$\cdot\mu$m^{-1}). We adjusted the viscosity of the yolk η_y. At each time step in the simulation, we computed the distance between the Animal and Vegetal poles and compared it with the experimental measurements (Figure S3K). This allowed us to obtain a value of the yolk viscosity equal to $\eta_y = 40$ Pa$\cdot$s, around 24 times smaller than the one obtained for the blastoderm viscosity (Table S1C).
