# Supplementary figures and images for: The Physical Basis of Coordinated Tissue Spreading in Zebrafish Gastrulation
Source: Dev Cell. 2017 Feb 27;40(4):354–366.e4. doi: 10.1016/j.devcel.2017.01.010 (PMC5364273; doi:10.1016/j.devcel.2017.01.010)

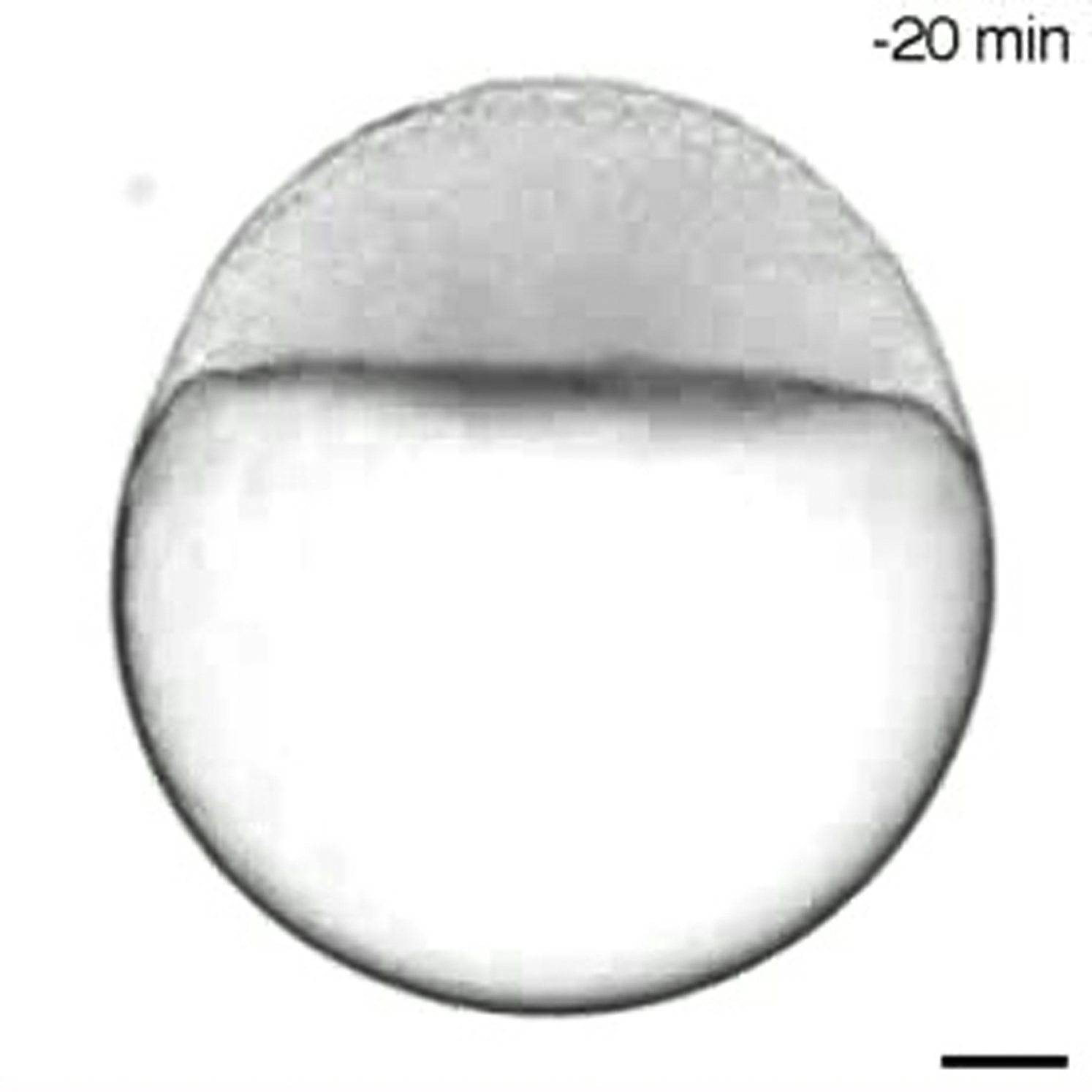

Supplement: Movie S1. Embryo Doming, Related to Figure 1 [file mmc3.jpg]

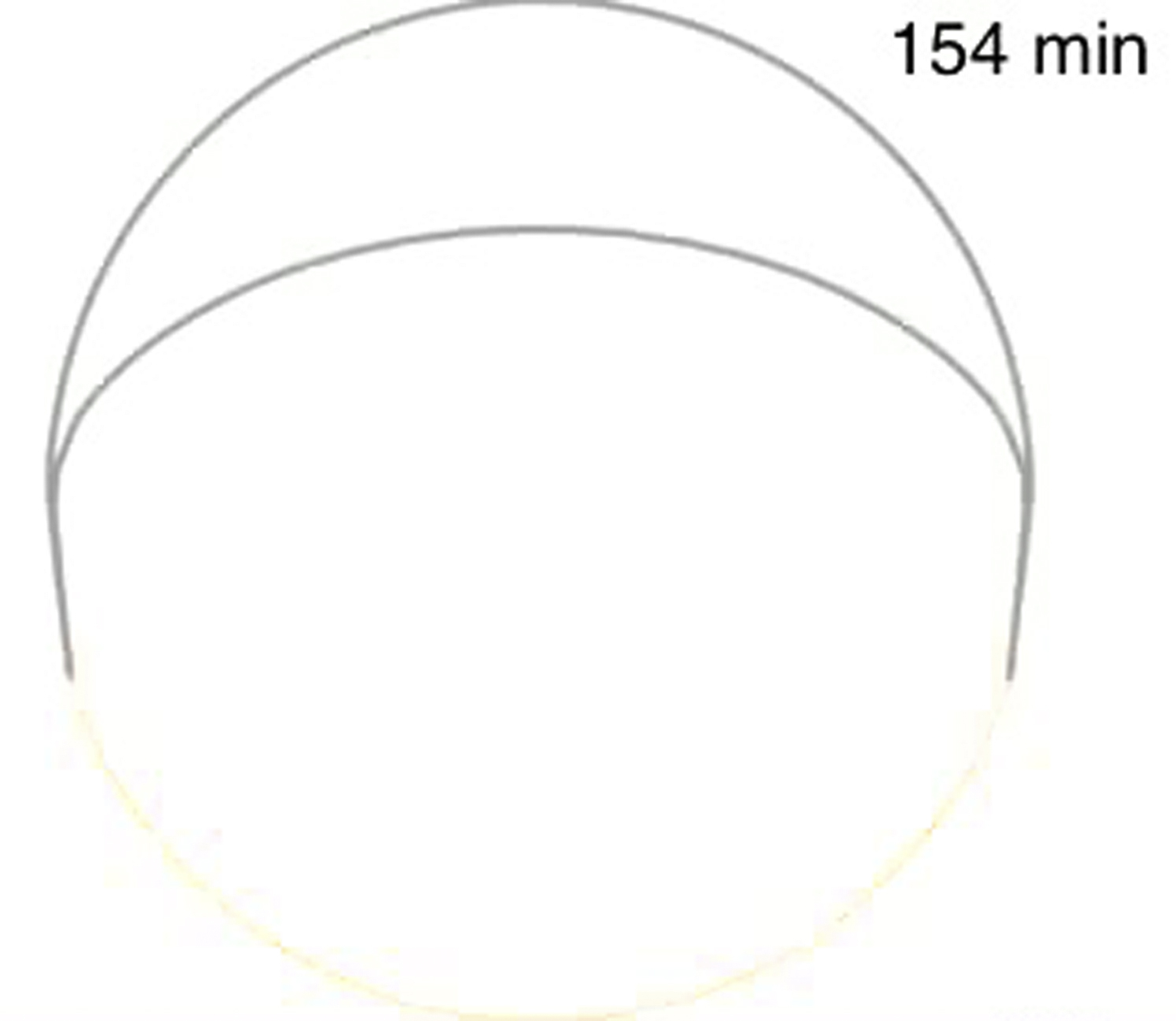

Supplement: Movie S2. Simulations of Embryo Doming, Related to Figure 2 [file mmc4.jpg]

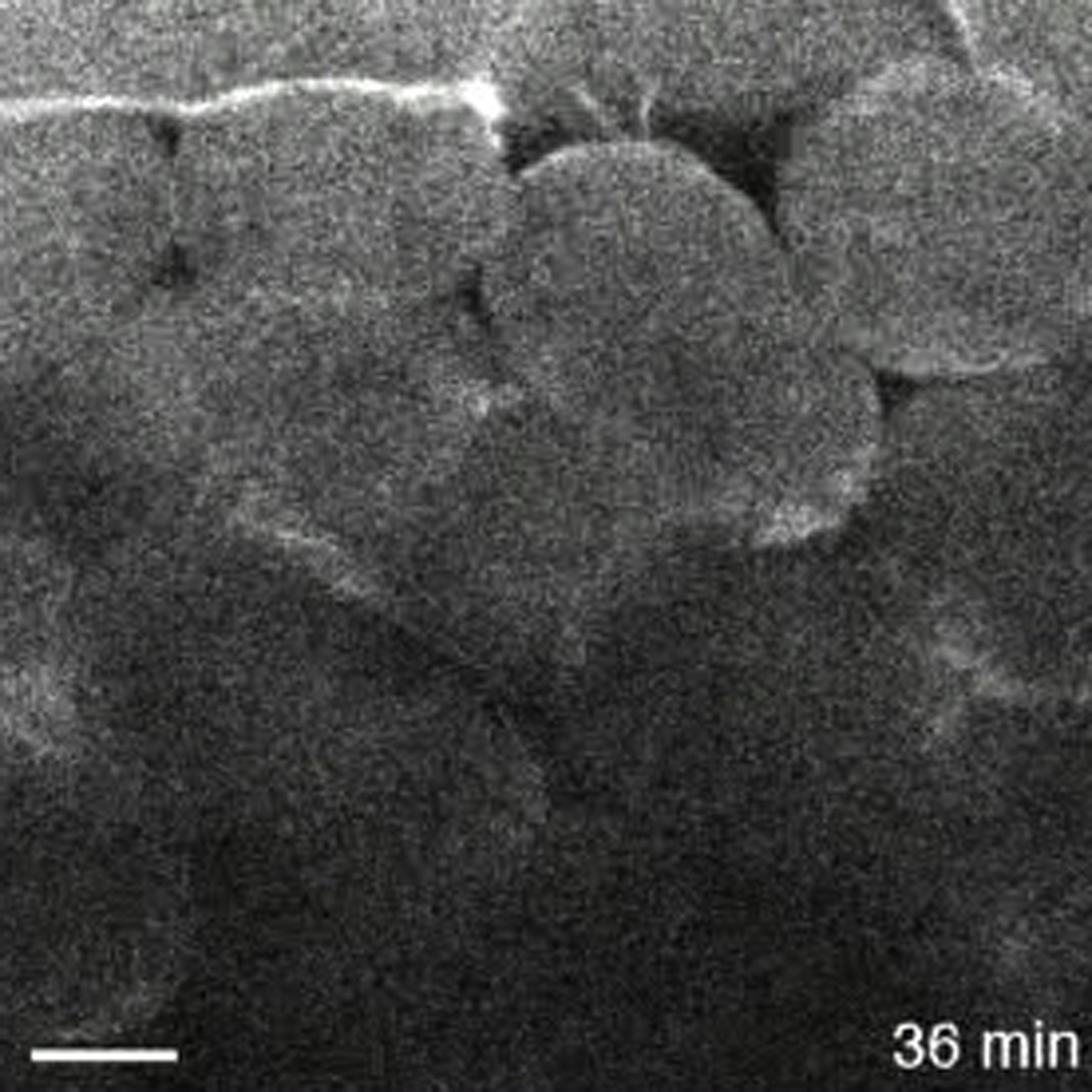

Supplement: Movie S3. Deep Cell Movesment and Polarization during Doming, Related to Figure 3 [file mmc5.jpg]

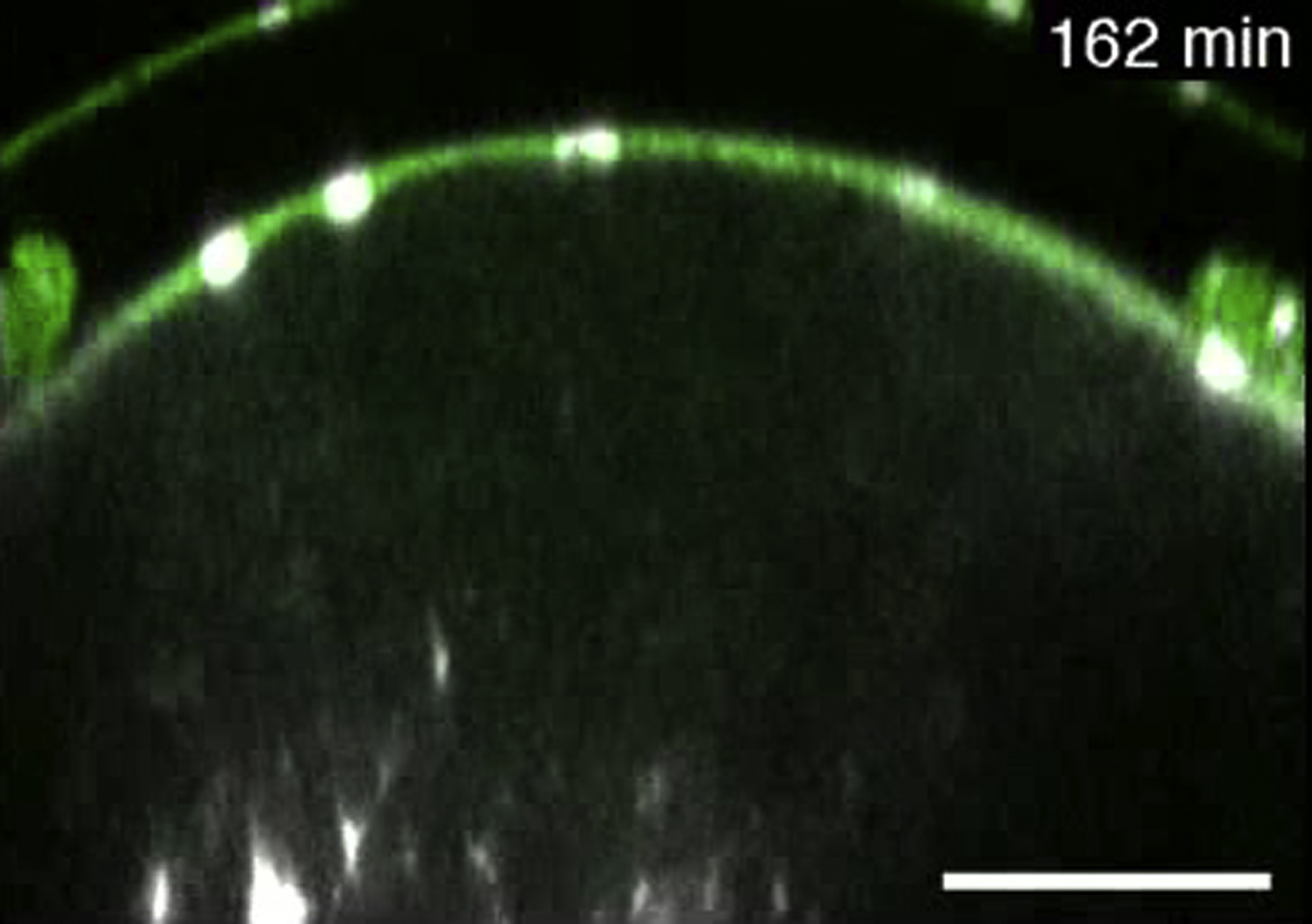

Supplement: Movie S4. Doming of Deep Cell-Depleted Embryo, Related to Figure 4 [file mmc6.jpg]

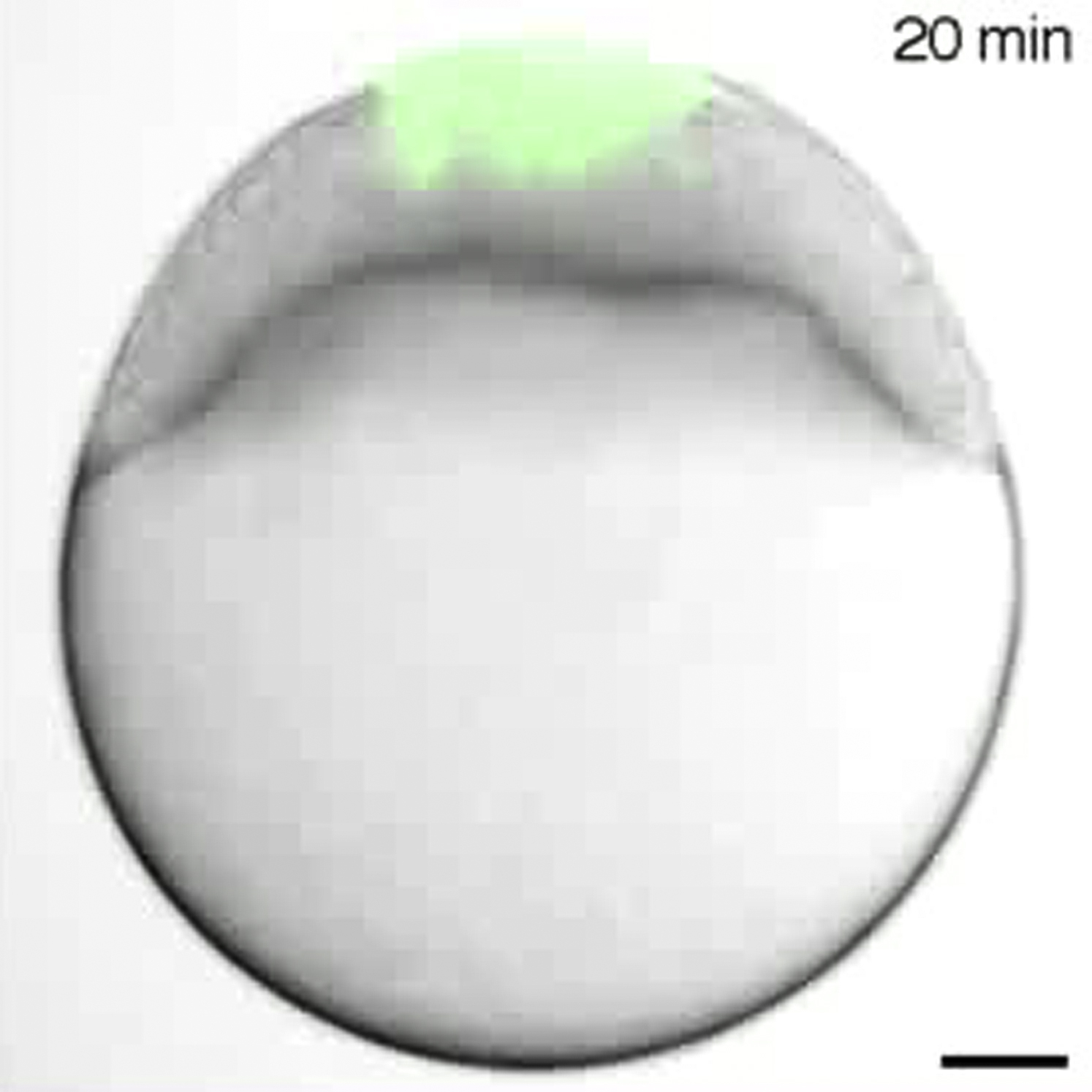

Supplement: Movie S5. Doming in EVL/Surface Cell Transplanted Embryos, Related to Figures 5 and 7 [file mmc7.jpg]

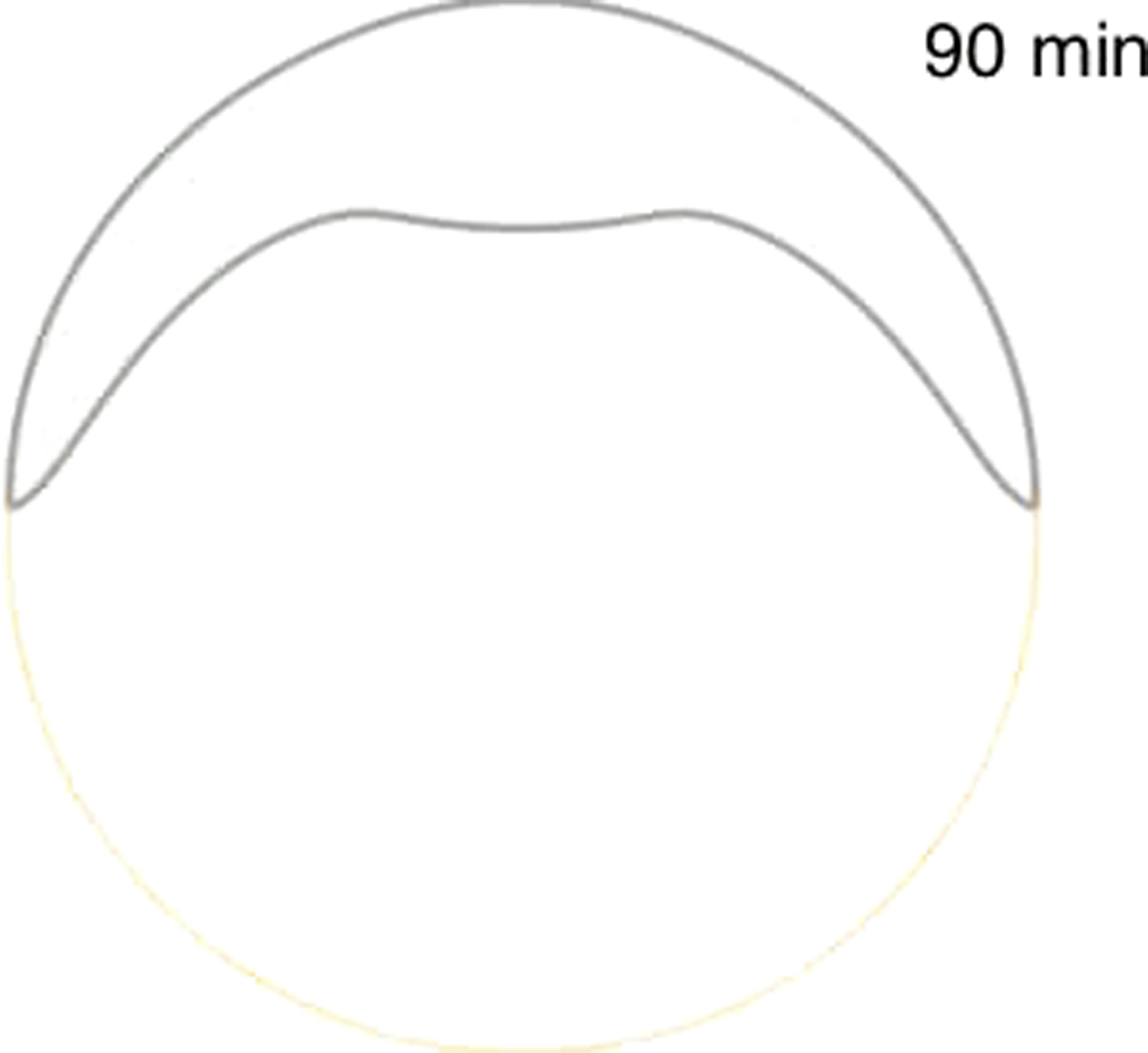

Supplement: Movie S6. Simulations of Doming in EVL/Surface Cell Transplanted Embryos, Related to Figure 6 [file mmc8.jpg]
